# Distribution and comparative genomic analysis of antimicrobial gene clusters found in *Pantoea*

**DOI:** 10.3389/fmicb.2024.1416674

**Published:** 2024-08-14

**Authors:** Ashlyn Kirk, John Stavrinides

**Affiliations:** Department of Biology, University of Regina, Regina, SK, Canada

**Keywords:** *Pantoea*, bacterial natural products, biosynthetic gene clusters, horizontal gene transfer, phylogenetics, comparative genomics, pantocin B, antibiotics

## Abstract

Members of the bacterial genus *Pantoea* produce a variety of antimicrobial products that are effective against plant, animal, and human pathogens. To date, little is known about the distribution and evolutionary history of these clusters. We surveyed the public databases for the 12 currently known antibiotic biosynthetic gene clusters found across *Pantoea* strains to determine their distribution. We show that some clusters, namely pantocin B, PNP-3, and PNP-4 are found strictly in *Pantoea,* while agglomerin, andrimid, AGA, dapdiamide, herbicolin, PNP-1, PNP-2, PNP-5, and pantocin A, are more broadly distributed in distantly related genera within *Vibrionaceae*, *Pectobacteriaceae*, *Yersiniaceae*, *Morganellaceae,* and *Hafniaceae*. We evaluated the evolutionary history of these gene clusters relative to a *cpn60*-based species tree, considering the flanking regions of each cluster, %GC, and presence of mobile genetic elements, and identified potential occurrences of horizontal gene transfer. Lastly, we also describe the biosynthetic gene cluster of pantocin B in the strain *Pantoea agglomerans* Eh318 more than 20 years after this antibiotic was first described.

## 1 Introduction

The genus *Pantoea* is a diverse group within the *Erwiniaceae* whose members inhabit a variety of aquatic and terrestrial environments (Walterson and Stavrinides, 2015). Members of *Pantoea* have also been found in association with various insects, animals, and humans (Walterson and Stavrinides, 2015). As a genus, *Pantoea* has a variety of unique characteristics and capabilities that make it of interest in both environmental and clinical settings including applications in biocontrol, bioremediation, biosensing, and therapeutics (Walterson and Stavrinides, 2015). Of significance, many *Pantoea* strains produce natural product antimicrobials effective against human, plant, and/or animal pathogens (Walterson and Stavrinides, 2015). These antimicrobials are primarily synthesized through secondary metabolic pathways via biosynthetic gene clusters (BGCs), which include genes that encode enzymes, as well as genes involved in regulation, export, and resistance. To date, there are at least 22 known unique antimicrobials found in members of *Pantoea*, which collectively represent 12 different BGCs.

Among the earliest described antimicrobials produced by *Pantoea* were the tetronate antibiotics, agglomerin A, B, C, and D, which were isolated from *Pantoea agglomerans* PB-6042 (Shoji et al., 1989). These compounds are synthesized by the same seven gene cluster and differ only in the structure of the hydrocarbon chain found on the acyl group of the compound (Terui et al., 1990; Kanchanabanca et al., 2013). The agglomerins show moderate activity against both Gram-positive and Gram-negative anaerobic bacteria (Shoji et al., 1989). Andrimid, another *Pantoea* antibiotic, is a pseudopeptide synthesized through a hybrid NRPS-PKS pathway (Jin et al., 2006). Andrimid was first extracted from the broth culture of a symbiont of a brown planthopper in 1987 (Fredenhagen et al., 1987); however, the 21-gene cluster responsible for the synthesis of andrimid was not described until almost 20 years later in *P. agglomerans* Eh335 (Jin et al., 2006). Andrimid acts by inhibiting the β-subunit of acetyl-CoA carboxylase in fatty acid synthesis, thus inhibiting cell growth (Freiberg et al., 2004; Jin et al., 2006). Andrimid is effective against both Gram-positive and Gram-negative pathogens, including methicillin-resistant *Staphylococcus aureus* and vancomycin-resistant *Enterococcus* (Needham et al., 1994).

*Pantoea* also produces phenazine antibiotics, most notably, D-alanylgriseoluteic acid (AGA). AGA was first described in *P. agglomerans* Eh1087 as inhibiting the growth of the phytopathogen, *Erwinia amylovora*, the causative agent of fire blight in apples and pears (Kearns and Hale, 1996; Kearns and Mahanty, 1998; Giddens et al., 2002). The spectrum of activity of AGA has since been expanded to include many Gram-positive bacteria including clinical strains of *S. pneumoniae* and methicillin-resistant *S. aureus* (Giddens and Bean, 2007). A 16-gene cluster is responsible for AGA biosynthesis and resistance, but also directs the synthesis of at least two phenazine intermediates: phenazine-1,6-dicarboxylic acid and griseoluteic acid (Giddens et al., 2002).

The tripeptide antibiotics, dapdiamide A, B, C, D, and E were first identified from *P. agglomerans* CU0119 (Dawlaty et al., 2010). These structurally similar compounds share a central L-2,3-diaminopropionic acid fragment, but differ in their two amide-linked side chains (Dawlaty et al., 2010). Dapdiamides A-E are synthesized by a nine-gene cluster (Dawlaty et al., 2010). The dapdiamide cluster is homologous to two other *Pantoea* antibiotic BGCs: herbicolin I produced by *Pantoea vagans* C9-1, and 2-amino-3-(oxirane-2,3-dicarboxamido)-propanoyl-valine (APV) produced by *P. agglomerans* 48b/90 (Völksch and Sammer, 2008; Smits et al., 2010, 2011; Kamber et al., 2012). Herbicolin I and APV are structurally identical to dapdiamide E, although the stereochemistry of these has not been confirmed (Völksch and Sammer, 2008; Smits et al., 2011; Kamber et al., 2012).

*Pantoea* strains have also been reported to produce the antifungals, herbicolin A and B. These two lipopeptides were first isolated from *P. agglomerans* A 111 (Winkelmann et al., 1981), and later from *P. agglomerans* ZJU23 (Xu et al., 2022), which led to the characterization of the BGC responsible for the production of both molecules and their mechanism of action. Herbicolin A is synthesized by a 10-gene cluster and is thought to inhibit sterol-containing fungi through the disruption of ergosterol-containing lipid rafts in fungal cell membranes (Xu et al., 2022). Herbicolin B is an intermediate of herbicolin A and is glycosylated by AcbI in the last steps of synthesis to form herbicolin A (Xu et al., 2022). Herbicolin B is also biologically active against sterol-containing fungi, but less so than herbicolin A (Greiner and Winkelmann, 1991; Xu et al., 2022).

The peptide antibiotics pantocin A and B, produced by *P. agglomerans* Eh318, were first described as inhibitors of *E. amylovora* (Wright et al., 2001). Pantocin A acts by inhibiting histidine biosynthesis, and thus, its activity is neutralized if exogenous histidine is available (Wright et al., 2001; Jin et al., 2003a). The BGC for pantocin A has been described as a three-gene operon approximately 2.5 kb in size (Jin et al., 2003b). A small open reading frame (ORF) directly upstream of the cluster, which encodes a 30-amino acid precursor peptide is also required for pantocin A biosynthesis (Jin et al., 2003b). The BGCs of two other previously described *Pantoea* antibiotics, the microcin MccEh252, produced by *P. agglomerans* Eh252, and herbicolin O, produced by *P. vagans* C9-1 share high sequence identity to the pantocin A BGC (Vanneste et al., 2008). Genetic and chemical analysis of herbicolin O has recently confirmed it to be the same antibiotic as pantocin A (Ishimaru et al., 2017). Some discrepancies between the physiochemical characteristics of MccEh252 and pantocin A have been reported; however, these may be due to differences in assaying methodologies (Vanneste et al., 2008). The range of activity of MccEh252 is also indistinguishable from that of pantocin A (Vanneste et al., 2008); thus, it is likely that these two antibiotics are identical. In contrast to pantocin A, pantocin B is arginine reversible and works to inhibit its target by disrupting arginine biosynthesis through inhibition of *N*-acetylornithine transaminase (Brady et al., 1999). Although the structure and mechanism of action are known for pantocin B (Brady et al., 1999), the BGC has yet to be published.

BGCs responsible for the production of *Pantoea* Natural Products (PNP) 1 through 5 have also been described, but little is known about their structures or modes of action (Walterson et al., 2014; Robinson et al., 2020; Williams et al., 2020; Williams and Stavrinides, 2020; Kirk and Stavrinides, 2023). PNP-1 from *Pantoea ananatis* BRT175 is encoded by an eight-gene cluster and is effective against *E. amylovora* and select *Pantoea* strains (Walterson et al., 2014). After the discovery of the PNP-1 gene cluster, the structure of PNP-1 was determined to be 4-formylaminooxyvinylglycine, a non-proteogenic amino acid belonging to the class of antibiotics known as the oxyvinylglycines (Okrent et al., 2018). The cluster for PNP-2, identified in the cystic fibrosis isolate *P. agglomerans* TX10 is composed of six genes (Robinson et al., 2020). The resulting antibiotic is broad-spectrum, with the ability to inhibit both Gram-positive and Gram-negative bacteria including *Enterobacter, Escherichia, Klebsiella, Kosakonia, Pseudocitrobacter, Salmonella, Staphylococcus,* and *Streptococcus* (Robinson et al., 2020). PNP-3 is also broad-spectrum, with the ability to inhibit drug-resistant strains of *Pseudomonas aeruginosa* and *Acinetobacter baumannii* in addition to *Klebsiella* *spp.*, *E. coli, Enterobacter* *spp.*, *S. aureus,* and *Streptococcus mutans* (Williams and Stavrinides, 2020). PNP-3 is encoded by an eight-gene cluster, and was found to be produced by *P. agglomerans* strains 3581 and SN01080 (Williams and Stavrinides, 2020).

The BGC for PNP-4 found in *P. agglomerans* B025670 consists of 14 genes and was identified using comparative genomic approaches (Williams et al., 2020). PNP-4 is effective against *Enterobacter* *spp.*, *E. amylovora*, *E. coli*, *Kosakonia* *spp.*, *Pseudocitrobacter* *spp.*, and *Salmonella Typhimurium*, including some MDR strains (Williams et al., 2020). Lastly, the cluster for PNP-5, found in the clinical isolate *P. agglomerans* 20KB447973, is composed of 10 genes and shares similarity to previously described gene clusters for the dithiolopyrrolone antibiotic, holomycin, found in *Streptomyces clavuligerus*, *Yersinia ruckeri*, and *Photobacterium galatheae* (Li and Walsh, 2010; Qin et al., 2013; Sheng-Da et al., 2021). PNP-5 has not yet been confirmed to be structurally identical to holomycin. PNP-5 shows activity against a variety of Gram-positive and Gram-negative bacteria including *Citrobacter* *spp.*, *E. hormaechei*, *Enterobacter* *spp.*, *E. amylovora*, *E. coli*, *Klebsiella* *spp.*, *L. lactis*, *Salmonella* Typhimurium, and *S. mutans* (Kirk and Stavrinides, 2023).

In this study, we determined the distribution of these 12 *Pantoea* antibiotic BGCs. We then created phylogenies to establish the evolutionary relationships between homologous clusters from different species and combined this with an analysis of the flanking regions to help assess the potential evolutionary history of each cluster. We show that some antibiotic gene clusters may represent more recent acquisitions, while other clusters that appear widely distributed among *Pantoea* strains may be older. We also delineate and describe the sequence of the pantocin B gene cluster from the strain *P. agglomerans* Eh318.

## 2 Materials and methods

### 2.1 Sequences and BLAST

The distributions of 12 antibiotic BGCs identified in *Pantoea* were assessed: agglomerin, andrimid, AGA, dapdiamide, herbicolin, PNP-1, PNP-2, PNP-3, PNP-4, PNP-5, pantocin A, and pantocin B (Table 1). BGCs were BLASTed against the non-redundant (nr) and whole-genome shotgun contigs (wgs) databases at NCBI^1^ using default blastn parameters (Supplementary Table 1). Standalone BLAST was also used for some incomplete and draft genomes using a word size of 11 with all other parameters set to default. Candidate BGCs that did not contain all of the genes found in the query cluster, or whose genes were not entirely syntenic relative to the query cluster were excluded. Also excluded were candidate BGCs that diverged by more than 60% nucleotide identity across the length of the query cluster. BGCs that met our thresholds, but were found across contigs in draft genomes were included in our analyses. The 5 kb flanking each end of each cluster was also extracted and ORFs were predicted using GeneMark.hmm with Heuristic Models (Besemer and Borodovsky, 1999). Clinker (Gilchrist and Chooi, 2021) was used to visualize clusters, and to identify homologs between clusters. Parameters were set to default, with a 30% amino acid identity cut-off for this analysis.

**Table 1:** Reference antibiotic biosynthetic gene clusters surveyed in this study.

Cluster	Strain	Length (bp)	Number of open reading frames	References	Accession number
Agglomerin	*P. agglomerans* PB-6042	8,009	7	Kanchanabanca et al. (2013)	HF565364.1
Andrimid	*P. agglomerans* Eh335	24,430	21	Jin et al. (2006)	AY192157.1
D-alanylgriseoluteic acid (AGA)	*P. agglomerans* Eh1087	14,924	16	Giddens et al. (2002)	AF451953.1
Dapdiamide	*P. agglomerans* CU0119	10,747	9	Dawlaty et al. (2010)	CP001894.1
Herbicolin	*P. agglomerans* ZJU23	42,666	10	Xu et al. (2022)	CP068441.1
Pantocin A	*P. agglomerans* Eh318	2,691	3	Jin et al. (2003b)	U81376.2
Pantocin B	*P. agglomerans* Eh318	17,288	13	Wright et al. (2006). This study.	AXOF01000035.1
*Pantoea* Natural Product 1 (PNP-1)	*P. ananatis* BRT175	9,537	8	Walterson et al. (2014)	ASJH01000002.1
*Pantoea* Natural Product 2 (PNP-2)	*P. agglomerans* TX10	5,784	6	Robinson et al. (2020)	MN329808.1
*Pantoea* Natural Product 3 (PNP-3)	*P. agglomerans* 3,581	8,487	8	Williams and Stavrinides (2020)	MN807451.1
*Pantoea* Natural Product 4 (PNP-4)	*P. agglomerans* B025670	14,327	14	Williams et al. (2020)	MT711882.1
*Pantoea* Natural Product 5 (PNP-5)	*P. agglomerans* 20 KB447973	Pending	10	Kirk and Stavrinides (2023)	Pending

### 2.2 Phylogenetic analysis

The nucleotide sequences of homologous gene clusters together with their intergenic regions were aligned with MUSCLE (Madeira et al., 2019) using default parameters. Maximum-likelihood trees for each gene cluster set were constructed with MEGAX (Stecher et al., 2020) using best-fit models (Supplementary Table 2) with 1,000 bootstrap replicates. Maximum-likelihood species trees were generated using available *cpn60* sequences for representative strains.

## 3 Results

### 3.1 Agglomerin

Homologs of the agglomerin cluster described from *P. agglomerans* PB-6042 were identified in a total of 181 strains, 178 of which belonged to the genus *Dickeya* (Table 2; Supplementary Tables 3, 4). No homologs were identified in any other strain of *Pantoea*. Neither the genome nor any barcoding regions for *P. agglomerans* PB-6042 are currently available to confirm the identity of this strain; consequently, the agglomerin cluster was not analyzed further.

**Table 2:** Distribution of *Pantoea* antibiotic biosynthetic gene clusters across representative genera.

Cluster	Genus	Number of strains containing cluster	Number of available genome projects and assemblies	Percent of strains containing cluster
Agglomerin	*Dickeya*	178	516	34.496%
	*Pantoea*	1	2,527	0.040%
	*Musicola*	2	6	33.333%
Andrimid	*Serratia*	27	6,933	0.389%
	*Pantoea*	11	2,527	0.435%
	*Vibrio*	2	56,817	0.004%
AGA	*Xenorhabdus*	11	354	3.107%
	*Pantoea*	10	2,527	0.396%
	*Pectobacterium*	10	1,182	0.846%
Dapdiamide	*Pantoea*	8	2,527	0.317%
	*Serratia*	1	6,933	0.014%
Herbicolin	*Pantoea*	3	2,527	0.119%
	*Candidatus* Fukatsuia symbiotica	1	2	50.000%
Pantocin A	*Pantoea*	47	2,527	1.860%
	*Dickeya*	5	516	0.969%
	Unknown *Pectobacteriaceae*	4	N/A	N/A
	*Edwardsiella*	3	241	1.245%
Pantocin B	*Pantoea*	3	2,527	0.119%
PNP-1	*Pantoea*	8	2,527	0.317%
	Unknown *Pectobacteriaceae*	1	N/A	N/A
PNP-2	*Enterobacter*	38	23,425	0.162%
	*Pantoea*	30	2,527	1.187%
	*Proteus*	27	4,919	0.549%
	*Serratia*	25	6,933	0.361%
	*Providencia*	18	2,273	0.792%
	*Pectobacterium*	10	1,182	0.846%
PNP-3	*Pantoea*	22	2,527	0.871%
PNP-4	*Pantoea*	14	2,527	0.554%
PNP-5	*Yersinia*	171	12,230	1.398%
	*Photobacterium*	13	975	1.333%
	*Serratia*	11	6,933	0.159%
	*Pantoea*	2	2,527	0.079%

### 3.2 Andrimid

The andrimid BGC was identified in 11 genomes from the nr database and 29 from the wgs (Supplementary Table 3). Of these 40 BGCs, 11 were found in *Pantoea*: six within *P. ananatis*, two within *P. stewartii*, one within *P. agglomerans,* and two within uncharacterized *Pantoea* strains (Supplementary Table 4). Homologs of the andrimid cluster were also found in members of *Serratia* and *Vibrio* (Supplementary Table 4). *Serratia* had the greatest number of andrimid homologs out of the three representative genera (Supplementary Table 4); however, there were almost three times the number of *Serratia* genome projects and assemblies available as compared to *Pantoea* (Table 2). A phylogenetic analysis of the andrimid cluster revealed two distinct lineages within each clade (Supplementary Figure 1A). The first lineage contained all *Pantoea* and *Vibrio* strains as well as some *Serratia* representatives, while the other contained only a subset of the *Serratia* representatives (Supplementary Figure 1A). The *cpn60* tree shows the expected relationships between representative taxa, with each species forming its own unique lineage (Supplementary Figure 1B).

Predicted ORFs flanking andrimid clusters were largely conserved within genera although there were some exceptions (Supplementary Figure 1C). The flanking region of the *P. agglomerans* Eh355 cluster was not conserved relative to the other *Pantoea* clusters, and there was also significant intra-species variation in the flanking regions of *S. plymuthica* andrimid clusters (Supplementary Figure 1C). One predicted ORF encoding a LysR-family transcriptional regulator was found upstream of all *Pantoea* and *Serratia* andrimid clusters, but not upstream of the *Vibrio* BGCs (Supplementary Figure 1C; orange arrow). Similarly, directly upstream of the andrimid cluster, a hypothetical protein-encoding gene was predicted in both *Vibrio* strains, most *Serratia,* and *P. agglomerans* Eh335 (Supplementary Figure 1C; green arrow). Interestingly, the last gene in the published andrimid cluster, *admU,* a predicted transposase, was only found in the original strain, *P. agglomerans* Eh355 (Supplementary Figure 1C). An analysis of the %GC of the andrimid cluster from *P. agglomerans* Eh335 revealed a GC content of 46.87%, while the genome of *P. agglomerans* Eh335 had a GC content of 54.56% (Table 3).

**Table 3:** %GC content of *Pantoea* antibiotic biosynthetic gene clusters and their host genomes/plasmids.

Cluster	%GC	Strain	%GC
Andrimid	46.87	*P. agglomerans* Eh335	54.56
AGA	43.72	*P. agglomerans* Eh1087	54.62
Herbicolin	59.72	*P. agglomerans* ZJU23 (plasmid unnamed 1)	54.06
Pantocin A	40.47	*P. agglomerans* Eh318	54.62
Pantocin B	35.15	*P. agglomerans* Eh318	54.62
PNP-1	44.23	*P. ananatis* BRT175	54.48
PNP-2	41.89	*P. agglomerans* TX10	54.63
PNP-3	49.06	*P. agglomerans* 3581	54.70
PNP-4	45.48	*P. agglomerans* B025670	54.85

### 3.3 D-alanylgriseoluteic acid

AGA cluster homologs were identified in 10 *P. agglomerans* strains, 10 *Pectobacterium* strains and 11 *Xenorhabdus* strains for a total of 31 AGA-containing representatives (Supplementary Tables 3, 4). AGA clusters were identified in a relatively small proportion of sequenced *Pantoea* genomes (0.4%) whereas it was distributed across 3% of available *Xenorhabdus* genomes (Table 2). The phylogeny of the AGA clusters formed three distinct lineages corresponding to the three genera, and this phylogeny was congruent with the *cpn60* tree (Supplementary Figures 2A,B). An analysis of the flanking regions of all *Pantoea* strains containing the AGA cluster revealed extensive conservation, except for potential frameshifts in the second predicted ORF upstream of the clusters and the third predicted ORF downstream of the clusters of *P. agglomerans* 190, CFSAN047154, CFSAN047153, Pa39-23, and Pa39-21 (Supplementary Figure 2C). Very few flanking regions of *Pectobacterium* AGA clusters were available, but those that could be analyzed exhibited little conservation between representatives (Supplementary Figure 2C). Flanking regions of BGCs identified in *Xenorhabdus* showed conservation within, but not across the two representative species, with *Xenorhabdus* sp. SF857 flanking regions found to be highly divergent in comparison to other *Xenorhabdus* strains (Supplementary Figure 2C). Interestingly, an approximately 500 bp ORF predicted to encode a DUF2165 family protein was identified downstream of the cluster across all representatives in all three genera, suggesting it may be important for AGA biosynthesis (Supplementary Figure 2C; purple arrow). Notably, the %GC of the *P. agglomerans* Eh1087 AGA cluster was over 10% lower than that of the genome (Table 3).

### 3.4 Dapdiamide

The dapdiamide cluster was found almost exclusively in *P. agglomerans,* with seven representatives carrying the cluster (Supplementary Tables 3, 4). Homologous clusters were also identified in both *P. vagans* and *Serratia inhibens* (Supplementary Table 4). A phylogenetic analysis of the dapdiamide clusters showed all *Pantoea* representatives grouping together (Supplementary Figure 3A), although *P. vagans* C9-1 nested within the *agglomerans* clusters (Supplementary Figures 3A,B). Further, *P. agglomerans* C410P1 and SI1_M5 formed sister taxa in the gene cluster tree, but not in the *cpn60* tree (Supplementary Figure 3B). The flanking regions surrounding the dapdiamide clusters showed the least amount of conservation in comparison to other antibiotic BGCs (Supplementary Figure 3C). Only one ORF predicted to encode a thioredoxin domain-containing protein directly downstream of the *Pantoea* clusters was found to be conserved, except in *P. agglomerans* DAPP-PG734 (Supplementary Figure 3C; green arrow). There were no shared ORFs between the flanking regions of *Pantoea* and the single *Serratia* representative.

### 3.5 Herbicolin

The sequence of the herbicolin cluster was obtained from the *P. agglomerans* ZJU23 genome (Xu et al., 2022). Our open-reading frame prediction resulted in two additional ORFs between *acbB* and *acbC* not previously described (Supplementary Figure 4C; Xu et al., 2022). These additional ORFs appeared to be the result of frameshift mutations in *acbC,* which may be sequencing artifacts. A search of the databases for this cluster identified it in three *Pantoea* strains and one *Candidatus* Fukatsuia symbiotica strain (Supplementary Tables 3, 4). It is unclear how widely distributed this cluster is across *Candidatus* F. symbiotica strains as there were only two genomes available; however, the herbicolin cluster appeared narrowly distributed across *Pantoea* (Table 2). Two *P. agglomerans* clusters were more closely related to each other than to the other two *Pantoea* representatives, which was consistent with the *cpn60* species tree (Supplementary Figures 4A,B). The ORFS upstream of the four BGCs were not conserved, although some of the genes downstream were conserved across the three *Pantoea* BGCs (Supplementary Figure 4C). The herbicolin cluster appears to be plasmid-encoded in *P. agglomerans* ZJU23, *P. agglomerans* 9Rz4 and *Candidatus* F. symbiotica 5D. The *P. agglomerans* ZJU23 herbicolin cluster had a 59.72% GC content, while its host plasmid had a GC content of 54.06% (Table 3).

### 3.6 Pantocin A

Fifty-nine strains were identified as carrying the pantocin A cluster, 47 of which were members of *Pantoea* and the remaining 12 were strains of *Dickeya chrysanthemi*, *Edwardsiella hoshinae,* and various members of the *Pectobacteriaceae* (Supplementary Table 4). Among members of *Pantoea*, the pantocin A cluster was most prevalent in *P. agglomerans* and *P. ananatis*, but it is also present in strains of *P. brenneri, P. vagans, P. stewartii,* and *Pantoea* *spp.* (Supplementary Table 4). The representative pantocin A clusters grouped into four distinct lineages that correspond to each genus, with the only exception being *D. chrysanthemi* Ech1591, which clustered with *Pantoea* strains instead of other *Dickeya* (Supplementary Figure 5A). The *Pantoea* lineage is subdivided into two well-supported lineages, one containing only *P. ananatis* and two *P. stewartii* strains, and the other all other representative *Pantoea* strains including one subset of *P. ananatis* strains (Supplementary Figure 5A). The antibiotic cluster tree was found to be mostly incongruent with the corresponding *cpn60* tree at the individual gene cluster level, although highly congruent at the genus level (Supplementary Figures 5A,B).

A comparison of the clusters identified in *Edwardsiella, Dickeya,* and the members of the *Pectobacteriaceae* showed that their flanking regions were mostly conserved, in contrast to those of *Pantoea*, which were variable (Supplementary Figure 5C). One ORF predicted to encode a hypothetical protein directly upstream of the cluster was found to be conserved across most *Pantoea* strains except for *P. agglomerans* Eh318, 540Y, Pa39-3, 20TX10122, Pa39-1, TX10, and PA4 (Supplementary Figure 5C; purple arrow). A predicted ABC transporter ATP-binding protein found upstream of the clusters of *P. ananatis* strains PNA_18_8S, PNA_18_9S, and PNA_18_10S was also identified upstream of the clusters of *Pectobacteriaceae* strains C52, C80, and CE70 and (Supplementary Figure 5C). The %GC content of *P. agglomerans* Eh318 was calculated to be 54.62%, while the pantocin A cluster from this strain was significantly lower at 40.47% GC (Table 3).

### 3.7 Pantocin B

At the time of our survey, the pantocin B gene cluster had not yet been fully described. To identify the BGC in the *P. agglomerans* Eh318 genome, we used a Perl script to search the available genome sequence for restriction site patterns that were previously reported (Wright et al., 2006). A single ~25 kb region with a nearly identical restriction site pattern was identified, which also matched the cluster size (~17 kb) and number of ORFs (13) that had been predicted previously (Supplementary Figure 6; Wright et al., 2006). Predicted protein products of ORFs in the pantocin B cluster based on BLASTx searches can be found in Supplementary Figure 6. Analysis of the distribution of the pantocin B gene cluster identified only two strains in *P. ananatis* that carry it in addition to *P. agglomerans* Eh318 (Supplementary Tables 3, 4; Supplementary Figure 7). The flanking regions of the cluster in the two *P. ananatis* strains were consistent with one another, although it is predicted that these strains may be clonal (Supplementary Figure 7C). The %GC content of the pantocin B cluster in *P. agglomerans* Eh318 was calculated to be almost 20% lower than that of the Eh318 genome (Table 3).

### 3.8 PNP-1

Eight homologous PNP-1 clusters were identified within *P. ananatis* and *P. stewartii* subsp. *indologenes* strains, along with one cluster in a strain belonging to a member of the *Pectobacteriaceae* (Supplementary Tables 3, 4). Clusters identified in *P. stewartii* formed a separate monophyletic group from those of *P. ananatis*, which was consistent with the relationship between these two species as shown in the *cpn60* tree (Supplementary Figures 8A,B). The *Pectobacteriaceae* bacterium CE90 cluster also formed its own unique lineage in the cluster phylogeny, consistent with the species tree (Supplementary Figures 8A,B). The flanking regions of the PNP-1 clusters were conserved across *Pantoea*, with some divergence seen upstream of the clusters between *P. ananatis* and *P. stewartii* subsp. *indologenes* strains, with some of the *P. ananatis* strains appearing to be clonal (i.e., PANS 99–36, PANS_99_36, and 99–36; PANS_99_25 and 99–25) (Supplementary Figures 8A–C). Flanking regions of *Pantoea* strains and the single *Pectobacteriaceae* representative were not conserved (Supplementary Figure 8C). Additionally, the %GC content of the PNP-1 cluster in *P. ananatis* BRT175 is approximately 10% lower than that of the BRT175 genome (Table 3).

### 3.9 PNP-2

The PNP-2 cluster was found to be broadly distributed, with homologs identified in 148 strains (Supplementary Tables 3, 4). The majority of *Pantoea* representatives containing the PNP-2 cluster were *P. agglomerans,* with others belonging to the species *P. vagans* and *P. pleuroti,* as well as unclassified *Pantoea* *spp.* (Supplementary Table 4). Almost half of the strains containing the PNP-2 cluster were non-*Pantoea* strains and included those from the genera *Providencia, Proteus, Pectobacterium, Enterobacter,* and *Serratia* (Supplementary Table 4). Proportionally, however, PNP-2 was found to be most broadly distributed within *Pantoea* (Table 2). Of note, homologs of *pnp2E*, a predicted ferredoxin reductase, in *Proteus* strains were truncated, with only the first portion of the gene being conserved (Supplementary Figure 9C).

A phylogenetic analysis of the PNP-2 BGC showed that clusters from each genus formed their own distinct lineages, with a few exceptions (Supplementary Figure 9A). First, *P. vagans* Mg1, *P. vagans* UBA6298, and *Pantoea* sp. S62 formed their own lineage separate from other *Pantoea* representatives, which was consistent with the *cpn60* tree (Supplementary Figures 9A,B). Second, several *E. asburiae* strains along with *P. agglomerans* T6 and SI1_M5 formed their own lineage separate from both genera (Supplementary Figure 9A). In contrast, these strains grouped with their respective species groups in the *cpn60* tree (Supplementary Figure 9B).

The flanking regions of the PNP-2 clusters identified in *Proteus* and *Providencia* were conserved in all representative strains, while the flanking regions of the *Enterobacter* clusters were conserved across all strains except for the six *E. asburiae* strains that were similar to each other (Supplementary Figure 9C). Similarly, the majority of *Pectobacterium* strains shared flanking regions, except for *Pe. colocasium* LJ1 and *Pectobacterium* sp. F1-1, which were more similar to each other, and *Pe. carotovorum* 251, which was unique (Supplementary Figure 9C). The most diversity in flanking regions was seen in *Serratia* and *Pantoea* (Supplementary Figure 9C). Additionally, the %GC of the PNP-2 cluster in *P. agglomerans* TX10 was significantly lower (41.89%) than that of the genome (Table 3).

### 3.10 PNP-3

The PNP-3 cluster was found only in members of *Pantoea* (Supplementary Tables 3, 4). Of the 22 strains, most were *P. agglomerans* and *P. vagans,* along with some strains identified as only *Pantoea* *spp.* (Supplementary Tables 3, 4). The PNP-3 cluster phylogeny formed three main lineages (Supplementary Figure 10A). The first lineage contained only *P. agglomerans,* the second contained *P. agglomerans* strains along with *P. vagans* C9-1, and the third contained only *Pantoea* *spp.* (Supplementary Figure 10A). Although *P. agglomerans* strains were not fully resolved in the *cpn60* tree, there was incongruence between the two trees, particularly within the second lineage, which grouped five *P. agglomerans* strains with the single *P. vagans* strain (Supplementary Figures 10A,B). Flanking regions of PNP-3 clusters showed high variability despite all representatives belonging to three *Pantoea* species (Supplementary Figure 10C). ORFs predicted to encode hypothetical proteins were found downstream of the cluster in all but four representatives (Supplementary Figure 10C; pink arrow). There were also several frameshift mutations identified within the PNP-3 cluster in several strains: *P. agglomerans* 553Y, 540Y, Pa31-4, Pa39-1, Pa39-7, Pa39-21, Pa39-23, *P. vagans* C9-1, *Pantoea* sp. M_6, M_8, and M_10 (Supplementary Figure 10C). The %GC of the *P. agglomerans* 3581 PNP-3 cluster is 49.06%, while that of the genome is 54.70% (Table 3).

### 3.11 PNP-4

The PNP-4 cluster was also restricted to *Pantoea*, but was distributed across multiple representatives of *P. dispersa, P. deleyi, P. ananatis, P. agglomerans,* and *Pantoea* *spp.* for a total of 14 representatives (Supplementary Tables 3, 4). Three distinct monophyletic groups emerged in the PNP-4 cluster tree (Supplementary Figure 11A), including one that comprised *P. agglomerans* strains as well as *Pantoea* *spp.* and *P. deleyi*, and the two others *P. dispersa* and *P. ananatis*, exclusively (Supplementary Figure 11A). The *cpn60* tree closely mirrored the cluster tree, except *P. deleyi* LMG24200, which formed its own lineage in the *cpn60* tree (Supplementary Figure 11B).

Flanking regions of PNP-4 clusters fell into four main patterns. The first group was composed of clusters from *P. deleyi* and *P. agglomerans* strains, and the second group comprised *Pantoea* sp. EKM21T and EKM22T, which had upstream genes that shared some similarity with the first group (Supplementary Figure 11C). *P. agglomerans* CFBP1316 was basal to these two groups and showed the same upstream ORF pattern as group one, but matched the downstream pattern of group 2 (Supplementary Figure 11C). Two *P. ananatis* strains made up group 3, while both *P. dispersa* strains as well as *P. agglomerans* M1657A made up group 4 (Supplementary Figure 11C). Neither group 3 nor group 4 shared flanking regions with the first two groups (Supplementary Figure 11C). The GC content of the *P. agglomerans* B025670 genome was approximately 10% higher than that of the PNP-4 cluster from this strain (45.48%) (Table 3).

### 3.12 PNP-5

The PNP-5 cluster was found to be distributed across 197 strains (Supplementary Tables 3, 4). Only two *Pantoea* species carry the cluster, *Pantoea* sp. 1.19 and *P. agglomerans* 20KB447973; however, previous work has reported that *Pantoea* sp. 1.19 may be misidentified (Kirk and Stavrinides, 2023). The remaining 195 clusters are found in members of *Serratia, Photobacterium,* and *Yersinia* (Supplementary Table 4). *Yersinia* strains accounted for 171 of the total representatives (Supplementary Table 4), which represented approximately 1.4% of all available *Yersinia* genomes (Table 2). The gene cluster tree is largely congruent to that of the species tree with respect to the monophyly of the representative genera; however, there were noticeable differences between the *Serratia* clades of the two trees (Supplementary Figures 12A,B).

The predicted ORFs in the flanking regions of PNP-5 clusters were conserved within genera, except *Serratia* sp. DD3, which did not show similarity to other *Serratia* representatives (Supplementary Figure 12C). *Photobacterium* strains showed the most diversity in flanking regions (Supplementary Figure 12C). Across most strains in *Photobacterium*, predicted ORFs for a LysR substrate-binding domain-containing protein and a DUF1127 domain-containing protein were found directly downstream of the cluster (Supplementary Figure 12C; green arrows). All *Photobacterium* strains also contained an additional gene within the PNP-5 cluster encoding a metallophosphoesterase (Supplementary Figure 12C; green arrow).

## 4 Discussion

We assessed the distribution of 12 known *Pantoea* antibiotic BGCs across public databases, and found that some clusters, namely pantocin B, PNP-3, and PNP-4 were found only in a relatively small subset of *Pantoea* strains. All other clusters had more broad distributions, encompassing members of *Pantoea* as well as other genera in the *Enterobacteriaceae, Pectobacteriaceae*, *Yersiniaceae*, *Morganellaceae,* and *Hafniaceae*. The andrimid and PNP-5 clusters also had representatives in the *Vibrionaceae*. In general, the andrimid, dapdiamide, pantocin A, PNP-1, and PNP-2 BGCs were more represented across *Pantoea* genomes than in any other genus, while the reverse was true for the AGA and PNP-5 BGCs (Table 2). This could suggest that the metabolites encoded by BGCs that are more prevalent across a diversity of *Pantoea* strains play a more central role in the general ecology of the group. Secondary metabolite BGCs are often reported to be reflective of ecotype or phylotype, with many gene clusters being species-specific (Jensen et al., 2007; Penn et al., 2009; Soucy et al., 2015; Xu et al., 2019). Still, inter-species and even inter-kingdom horizontal transfer of BGCs can occur (Vior et al., 2018; Kominek et al., 2019).

Our results are consistent with previous reports that many strains carry more than one BGC. *P. agglomerans* Pa31-3, Pa39-3, and Pa39-1 possess the BGCs for both pantocin A and PNP-3, *P. agglomerans* DAPP-PG734 has the BGCs for dapdiamide and PNP-4, and *P. agglomerans* strain 4 has the BGCs for dapdiamide, PNP-2 and PNP-3 (Sulja et al., 2022). Our previous work reported that *P. agglomerans* TX10 had clusters for both PNP-2 and pantocin A (Robinson et al., 2020) while *P. agglomerans* 3581 had the pantocin A cluster in addition to the PNP-3 cluster (Williams and Stavrinides, 2020). Our analysis also supported previous reports that *P. vagans* C9-1 has the BGCs for pantocin A, dapdiamide, and PNP-3 (Smits et al., 2011; Williams and Stavrinides, 2020; Sulja et al., 2022) and *P. agglomerans* Eh318 has the BGCs for pantocin A and B (Wright et al., 2001). In addition to these examples, we found several other strains that carry multiple BGCs. *P. agglomerans* CFSAN047153 and CFSAN047154 as well as *Pectobacterium* sp. F1-1 have both the AGA and PNP-2 clusters while *P. agglomerans* 39–23 and 39–7 have the AGA and PNP-3 clusters. *P. agglomerans* 540Y has both the Pantocin A and PNP-3 clusters, *P. agglomerans* Sl1_M5 has the dapdiamide and PNP-2 clusters, and *P. agglomerans* 9Rz4 has both the PNP-3 cluster as well as the herbicolin cluster. *P. agglomerans* VRA_MhP_f has both the PNP-2 and PNP-3 clusters. *Pectobacteriaceae* bacterium CE90 also contains two clusters: pantocin A and PNP-1. While our survey provides a glimpse into the distribution of these BGCs across strains, whether all these gene clusters are expressed and produce a bioactive metabolite remains unclear.

Our survey only focused on BGCs that shared significant sequence identity to the original reference cluster. For example, the PNP-1 cluster as described in *P. ananatis* BRT175 is composed of eight genes and produces 4-formylaminooxyvinylglycine, whereas the gene cluster in *P. fluorescens* that produces 4-formylaminooxyvinylglycine is composed of 11 genes (Walterson et al., 2014; Okrent et al., 2018). Experimental validation was needed to establish that these two BGCs produce a similar metabolite; consequently, we opted to use more stringent criteria and thresholds for this analysis. Expanding the survey to include other BGCs that share most or all of the genes of the reference BGCs would provide unique insight into the step-wise evolution of these BGCs.

Given the roles of natural product BGCs in competition and niche-specific adaptation, they are often found associated with mobile genetic elements (Ziemert et al., 2014; Vior et al., 2018). Potential horizontal gene transfer (HGT) events can be identified by comparing the evolutionary history of the BGCs to that of the host strains, with congruence indicating vertical transmission (Matter et al., 2009; Ziemert et al., 2014). For our analysis we used the barcoding gene *cpn60,* which can efficiently establish phylogenetic relationships between bacteria at the species level (Verbeke et al., 2011). We also constructed phylogenies using entire antimicrobial gene clusters, which helps to offset any strong phylogenetic signal from individual genes that may be evolving at different rates (Ziemert et al., 2014). Several instances of potential HGT were seen when comparing these trees to their sister *cpn60* trees. For example, in the dapdiamide cluster tree, *P. agglomerans* DAPP-PG734 did not group with other *P. agglomerans* strains as seen in the *cpn60* tree (Supplementary Figures 3A,B), suggesting the cluster has a unique evolutionary history in comparison to host strain. Analysis of the flanking regions of *P. agglomerans* DAPP-PG734 supports evolution through horizontal transfer, as it does not share homologous genes with any other *Pantoea* representatives (Supplementary Figure 3C). The flanking regions of the cluster in DAPP-PG734 also contain remnants of transposase genes, which are often associated with HGT events. In addition, the dapdiamide clusters found in *P. vagans* C9-1 and *P. agglomerans* C410P1, DAPP-PG734 and Sl1_M5 are predicted to be plasmid-encoded, and in some cases are adjacent to plasmid-associated genes such as the conjugation-associated gene, *traI* (Supplementary Figure 3C; dark blue arrow). The association of BGCs with mobile genetic elements can also be seen in strains carrying the andrimid, pantocin A, PNP-2, PNP-3, and PNP-4 clusters.

Although only some BGCs were directly associated with mobile genetic elements, our %GC analysis indicated that most BGCs have 5–20% lower %GC relative to their parent genome or plasmid, with the exception of herbicolin BGC, which has a %GC approximately 5% higher than the genome (Table 3). The herbicolin cluster is found in *Candidatus* F. symbiotica 5D, a symbiont of pea aphids (Patel et al., 2019), many of which provide their hosts with antifungal defenses (Łukasik et al., 2013). Given the narrow distribution of this cluster, it is interesting that these two relatively distant species share this cluster. As previously suggested, HGT may have occurred between *Pantoea* and *Candidatus* F. symbiotica (Xu et al., 2022), which is the most parsimonious explanation given the current data. Similarly, HGT may account for the PNP-1 cluster being common to both *Pantoea* and the unknown *Pectobacteriaceae* strain, as no other closely related strains have been identified that carry the cluster.

Our survey provides a comprehensive assessment of the distribution and potential evolutionary histories of known *Pantoea* antibiotic BGCs to date. We show these clusters have very different distributions, ranging from more restricted to broadly distributed across distantly related families. Some of these distributions, coupled with the presence of mobile genetic elements and analyses of %GC are suggestive of HGT. An understanding of the roles of the metabolites produced by these BGCs will provide much needed insight into the ecology and evolution of *Pantoea* and other closely related taxa.

## Data Availability

The original contributions presented in the study are included in the article/Supplementary material, further inquiries can be directed to the corresponding author.

## Footnotes

^1^ncbi.nlm.nih.gov; accessed on October 20, 2023.
