# Supplementary material for: Distribution and comparative genomic analysis of antimicrobial gene clusters found in Pantoea
Source: Front Microbiol. 2024 Aug 14;15:1416674. doi: 10.3389/fmicb.2024.1416674 (PMC11350110; doi:10.3389/fmicb.2024.1416674)
Supplement: Supplementary file 1 [file Data_Sheet_1.PDF]

# SUPPLEMENTARY MATERIAL

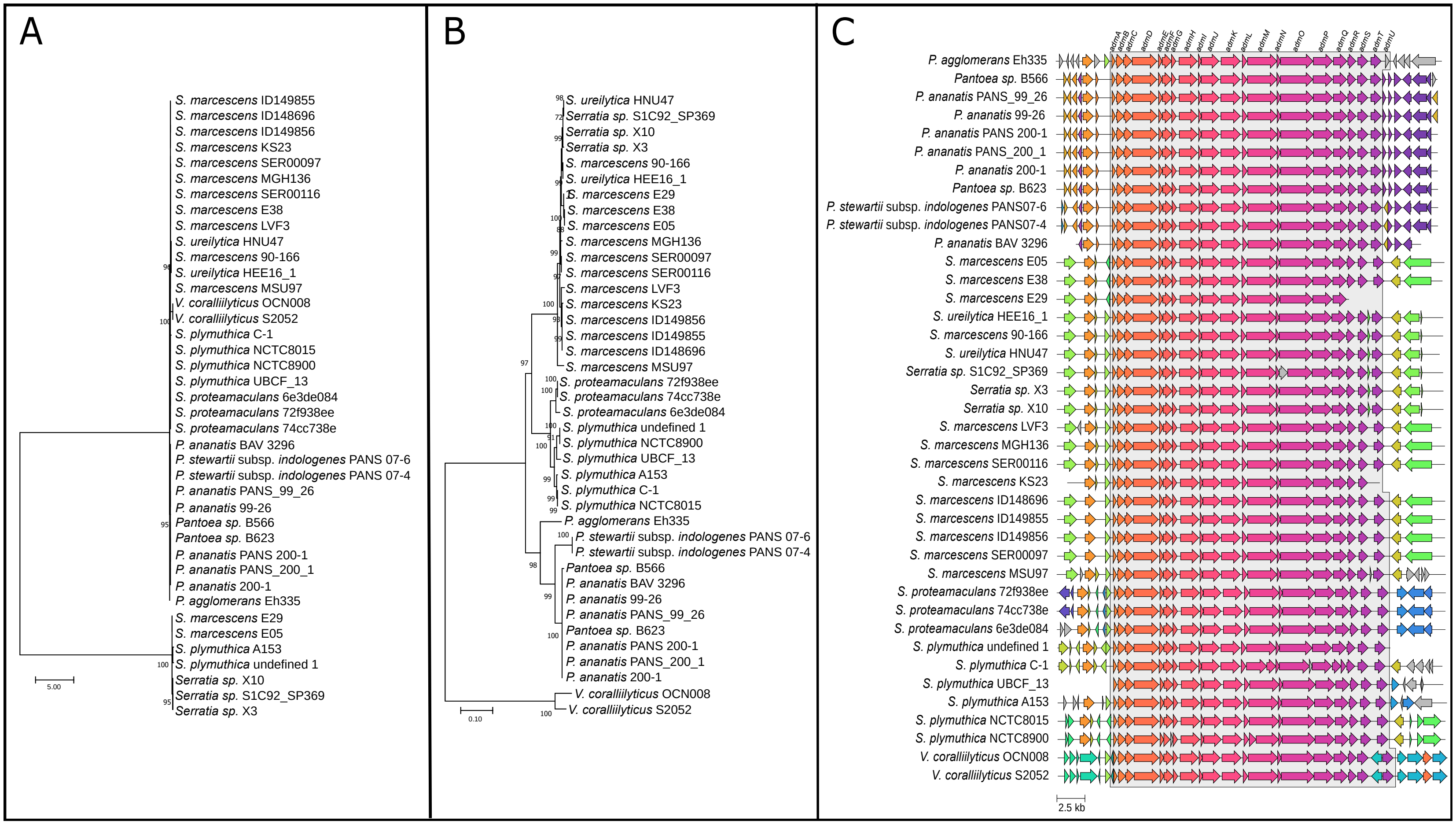


**Supplementary Figure 1.** Phylogenetic and comparative genomic analysis of andrimid clusters identified across bacterial strains. Panel A shows the maximum likelihood tree of andrimid clusters from representative strains, while panel B shows the maximum likelihood *cpn60* tree of strains carrying the andrimid cluster. Both trees were constructed using 1000 bootstrap replicates. Panel C shows the ORFs of each cluster (shown enclosed in the grey box) along with the genes flanking each end as predicted using GeneMark.hmm with Heuristic Models (Besemer and Borodovsky, 1999). Gene maps were generated with Clinker (Gilchrist and Chooi, 2021)

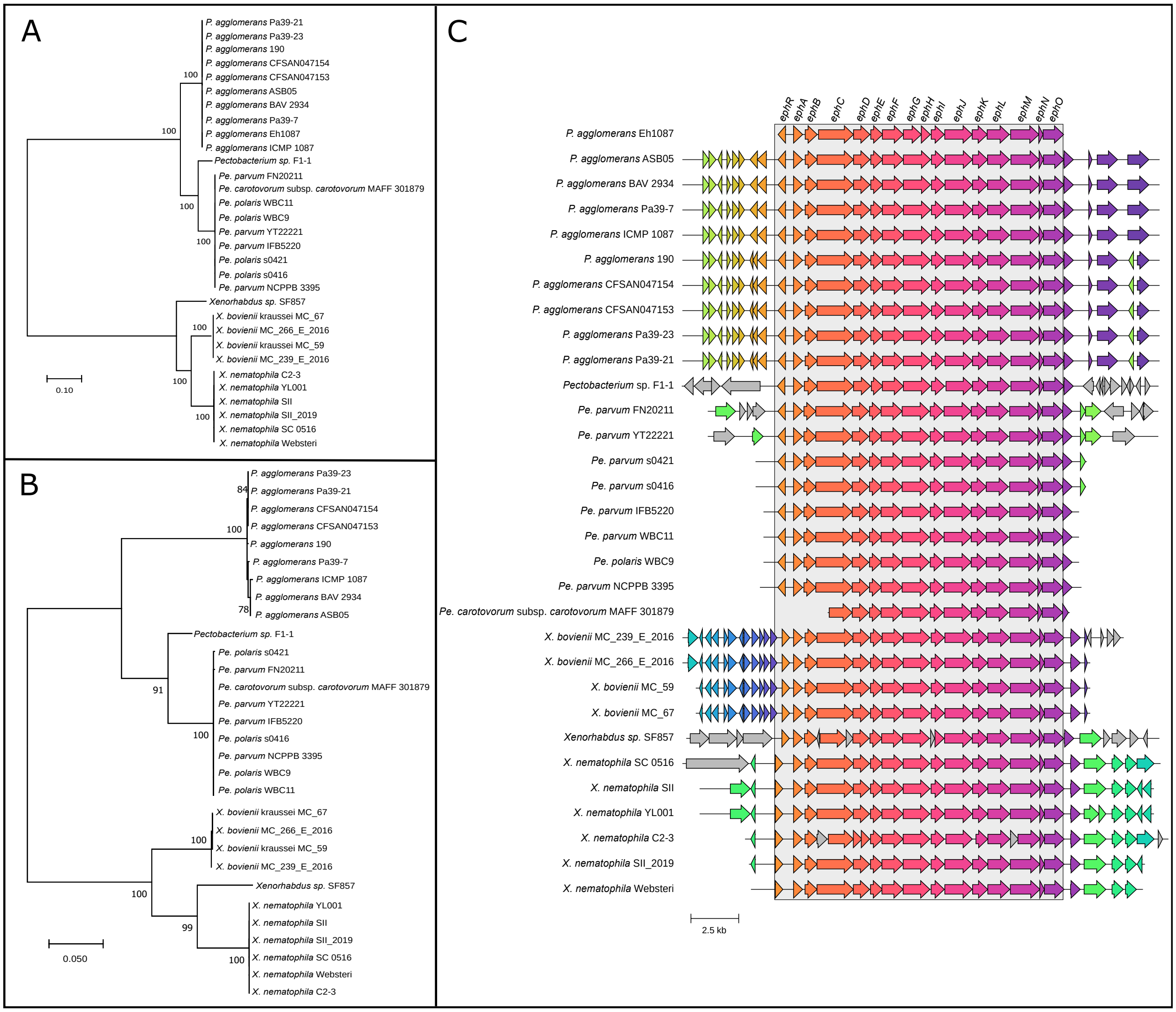


**Supplementary Figure 2.** Phylogenetic and comparative genomic analysis of D-Alanylgriseoluteic Acid (AGA) clusters identified across bacterial strains. Panel A shows the maximum likelihood tree of AGA clusters from representative strains, while panel B shows the maximum likelihood *cpn60* tree of strains carrying the AGA cluster. Both trees were constructed using 1000 bootstrap replicates. Panel C shows the ORFs of each cluster (shown enclosed in the grey box) along with the genes flanking each end as predicted using GeneMark.hmm with Heuristic Models (Besemer and Borodovsky, 1999). Gene maps were generated with Clinker (Gilchrist and Chooi, 2021).

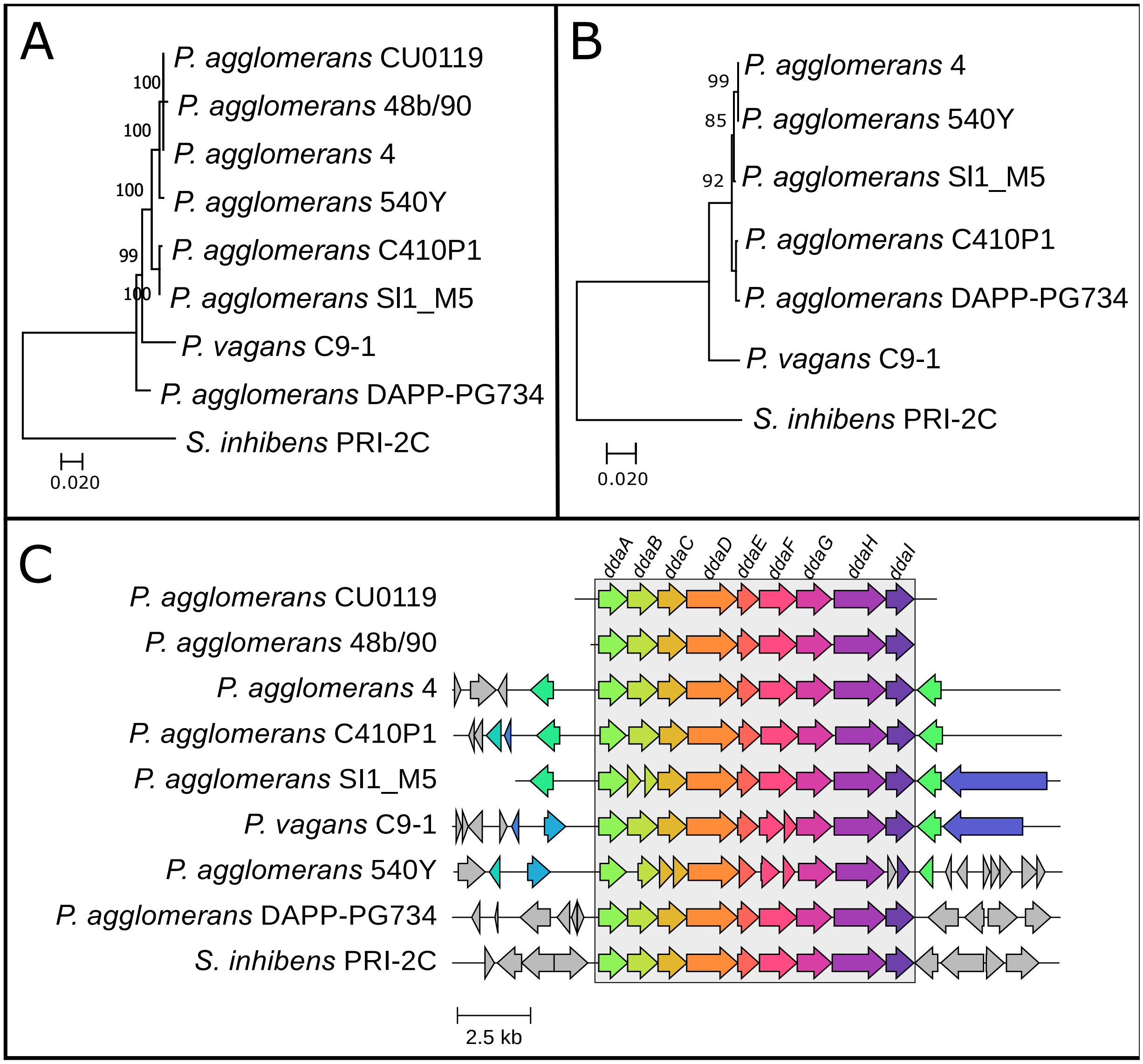


**Supplementary Figure 3.** Phylogenetic and comparative genomic analysis of dapdiamide clusters identified across bacterial strains. Panel A shows the maximum likelihood tree of dapdiamide clusters from representative strains, while panel B shows the maximum likelihood *cpn60* tree of strains carrying the dapdiamide cluster. Both trees were constructed using 1000 bootstrap replicates. Panel C shows the ORFs of each cluster (shown enclosed in the grey box) along with the genes flanking each end as predicted using GeneMark.hmm with Heuristic Models (Besemer and Borodovsky, 1999). Gene maps were generated with Clinker (Gilchrist and Chooi, 2021).

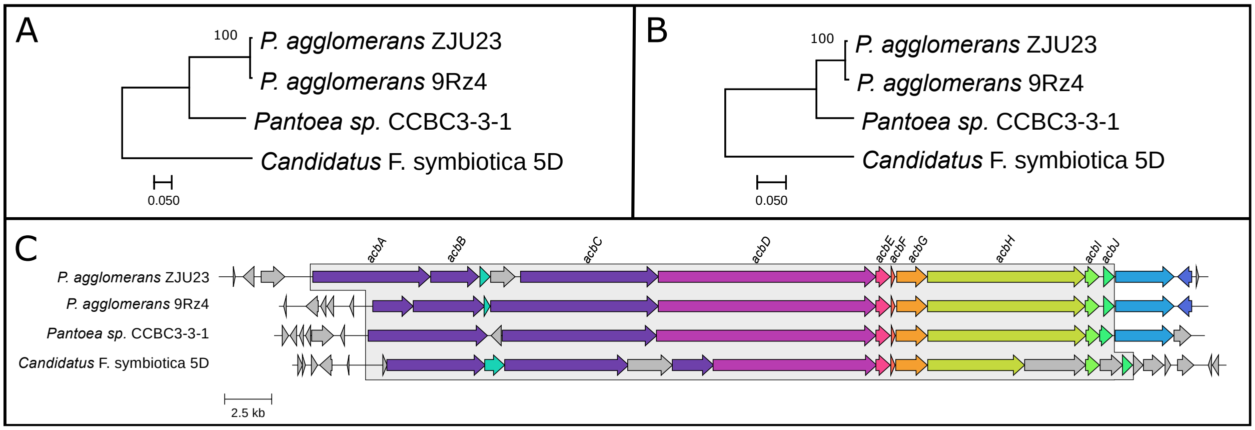


**Supplementary Figure 4.** Phylogenetic and comparative genomic analysis of herbicolin clusters identified across bacterial strains. Panel A shows the maximum likelihood tree of herbicolin clusters from representative strains, while panel B shows the maximum likelihood *cpn60* tree of strains carrying the herbicolin cluster. Both trees were constructed using 1000 bootstrap replicates. Panel C shows the ORFs of each cluster (shown enclosed in the grey box) along with the genes flanking each end as predicted using GeneMark.hmm with Heuristic Models (Besemer and Borodovsky, 1999). Gene maps were generated with Clinker (Gilchrist and Chooi, 2021).

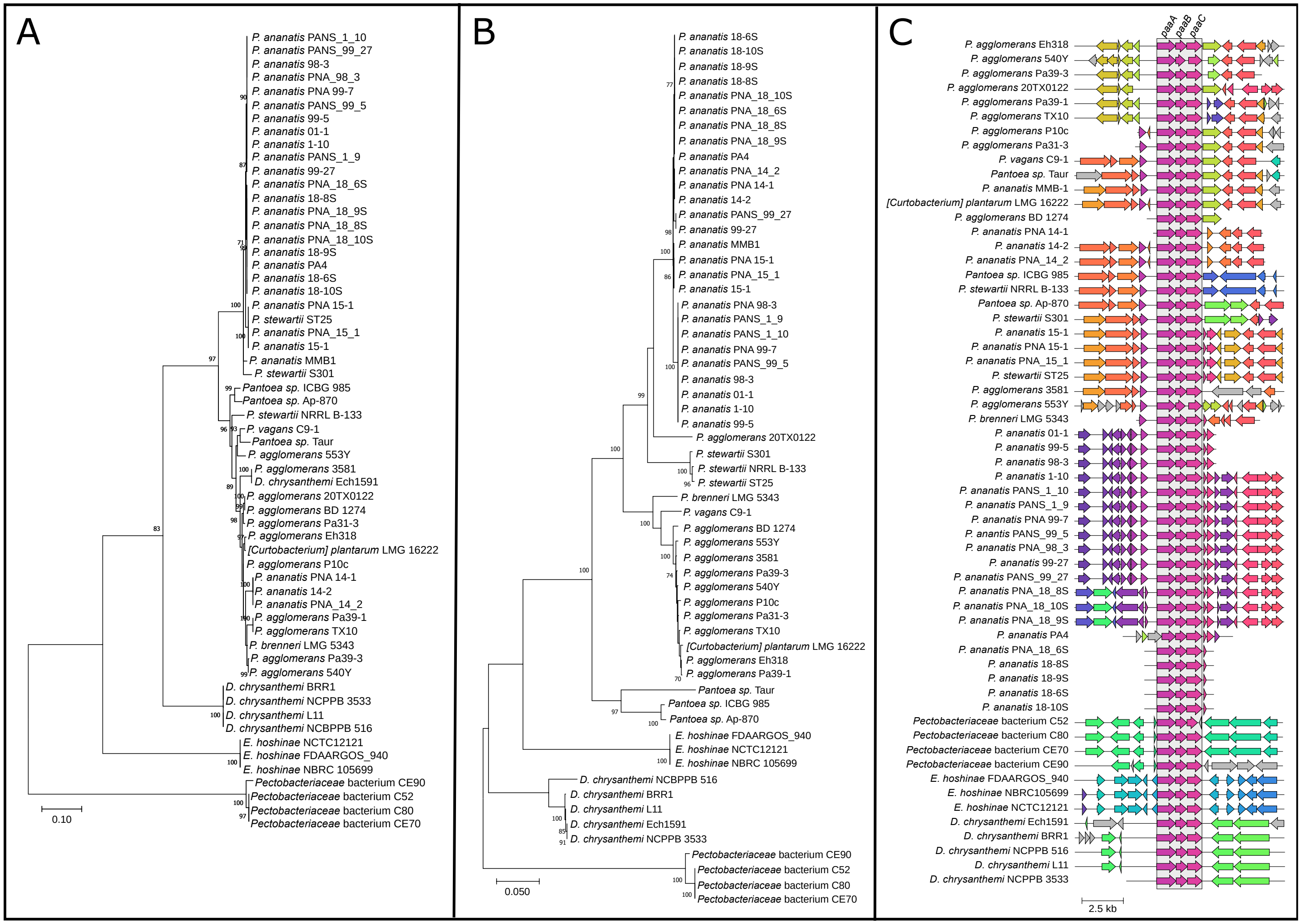


**Supplementary Figure 5.** Phylogenetic and comparative genomic analysis of pantocin A clusters identified across various bacterial strains. Panel A shows the maximum likelihood tree of pantocin A clusters from representative strains, while panel B shows the maximum likelihood *cpn60* tree of strains containing the pantocin A cluster. Both trees were constructed using 1000 bootstrap replicates. Panel C shows the ORFs of each cluster (shown enclosed in the grey box) along with the genes flanking each end as predicted using GeneMark.hmm with Heuristic Models (Besemer and Borodovsky, 1999). Gene maps were generated with Clinker (Gilchrist and Chooi, 2021)

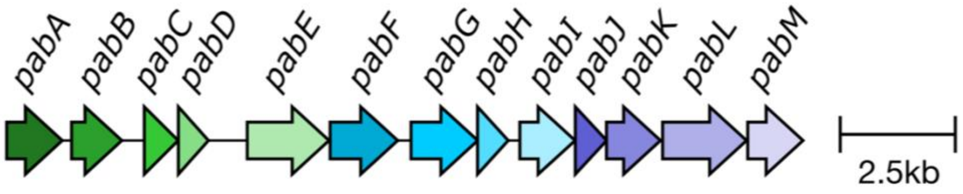


| Gene | Predicted Protein Product |
|---|---|
| *pabA* | MFS transporter |
| *pabB* | C45 family peptidase |
| *pabC* | Gamma-glutamyl-gamma-aminobutyrate hydrolase family protein |
| *pabD* | GNAT family N-acetyltransferase |
| *pabE* | FAD-dependent oxidoreductase |
| *pabF* | Pyridoxal-dependent decarboxylase |
| *pabG* | Amidase |
| *pabH* | Class I SAM-dependent methyltransferase |
| *pabI* | ATP-grasp domain containing protein |
| *pabJ* | Glutathione S-transferase |
| *pabK* | PLP-dependent aspartate aminotransferase family protein |
| *pabL* | FAD/NAD(P)-binding protein |
| *pabM* | CoA transferase |

**Supplementary Figure 6.** Gene map and predicted protein products of the pantocin B cluster.

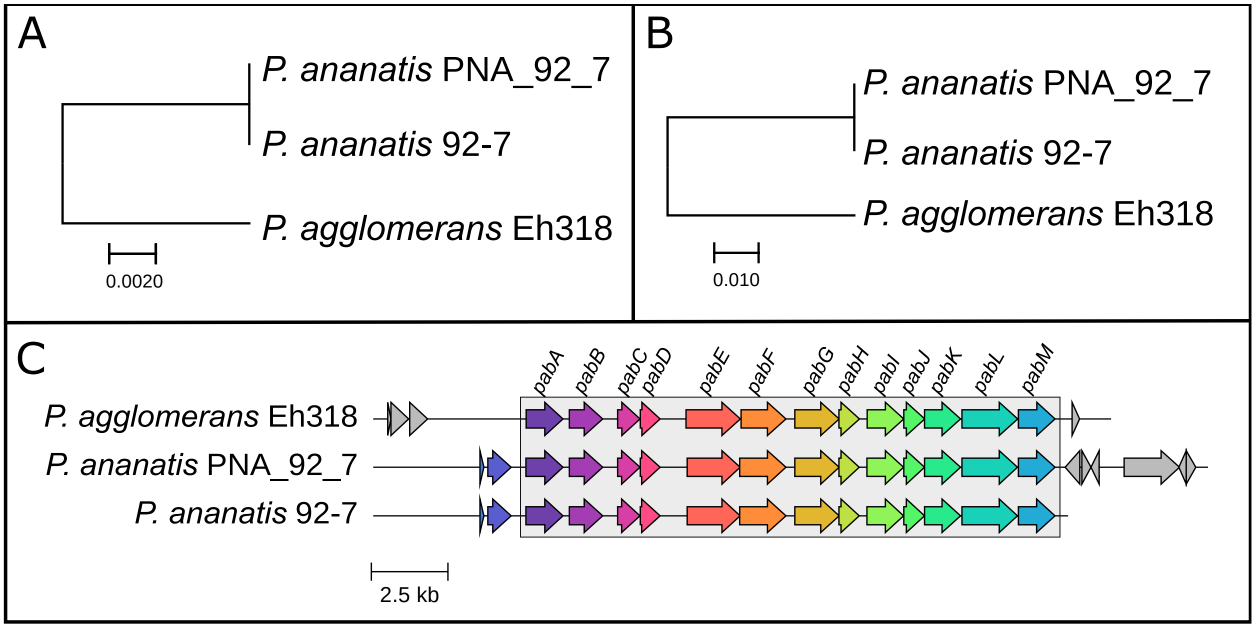


**Supplementary Figure 7.** Phylogenetic and comparative genomic analysis of pantocin B clusters identified across bacterial strains. Panel A shows the maximum likelihood tree of pantocin B clusters from representative strains, while panel B shows the maximum likelihood *cpn60* tree of strains carrying the pantocin B cluster. Panel C shows the ORFs of each cluster (shown enclosed in the grey box) along with the genes flanking each end as predicted using GeneMark.hmm with Heuristic Models (Besemer and Borodovsky, 1999). Gene maps were generated with Clinker (Gilchrist and Chooi, 2021).

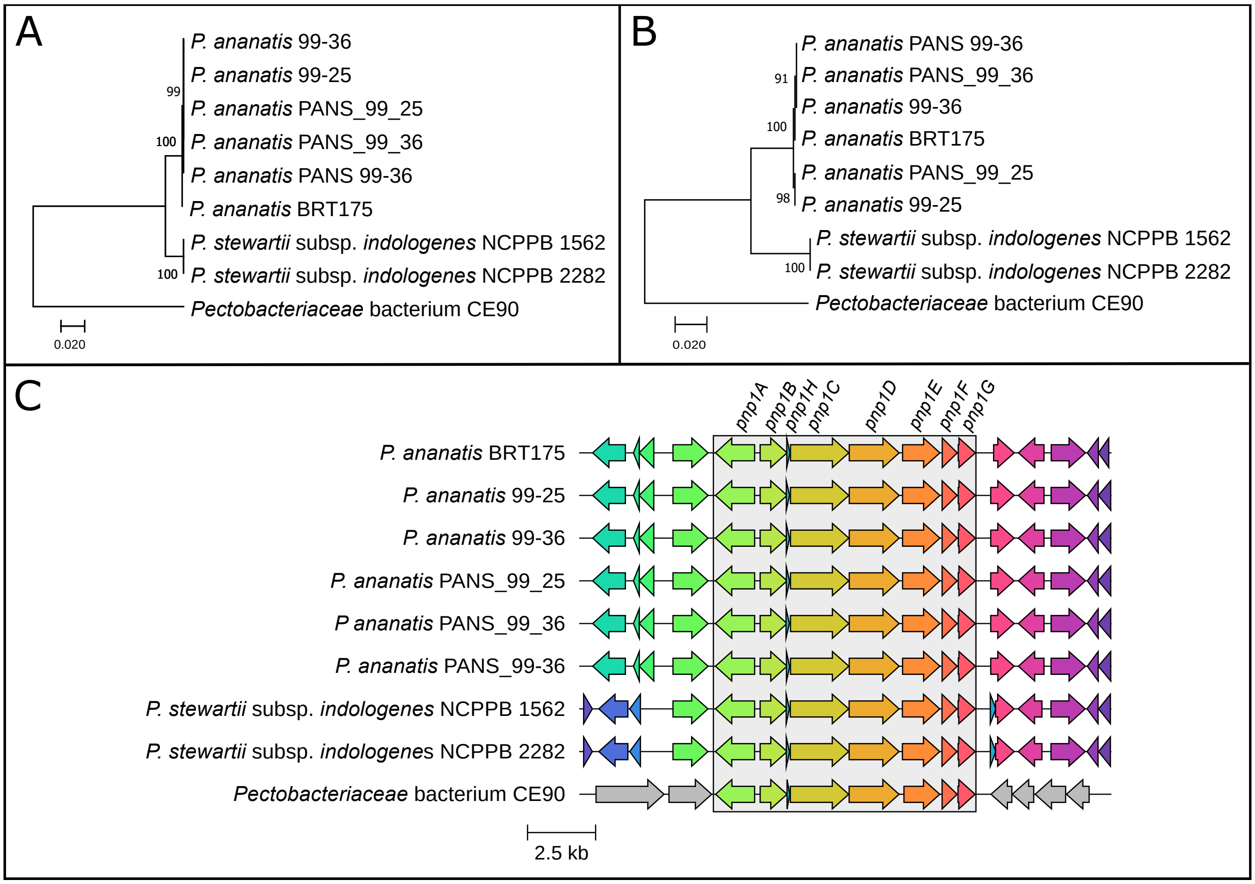


**Supplementary Figure 8.** Phylogenetic and comparative genomic analysis of *Pantoea* natural product 1 (PNP-1) clusters identified across bacterial strains. Panel A shows the maximum likelihood tree of PNP-1 clusters from representative strains, while panel B shows the maximum likelihood *cpn60* tree of strains carrying the PNP-1 cluster. Both trees were constructed using 1000 bootstrap replicates. Panel C shows the ORFs of each cluster (shown enclosed in the grey box) along with the genes flanking each end as predicted using GeneMark.hmm with Heuristic Models (Besemer and Borodovsky, 1999). Gene maps were generated with Clinker (Gilchrist and Chooi, 2021).

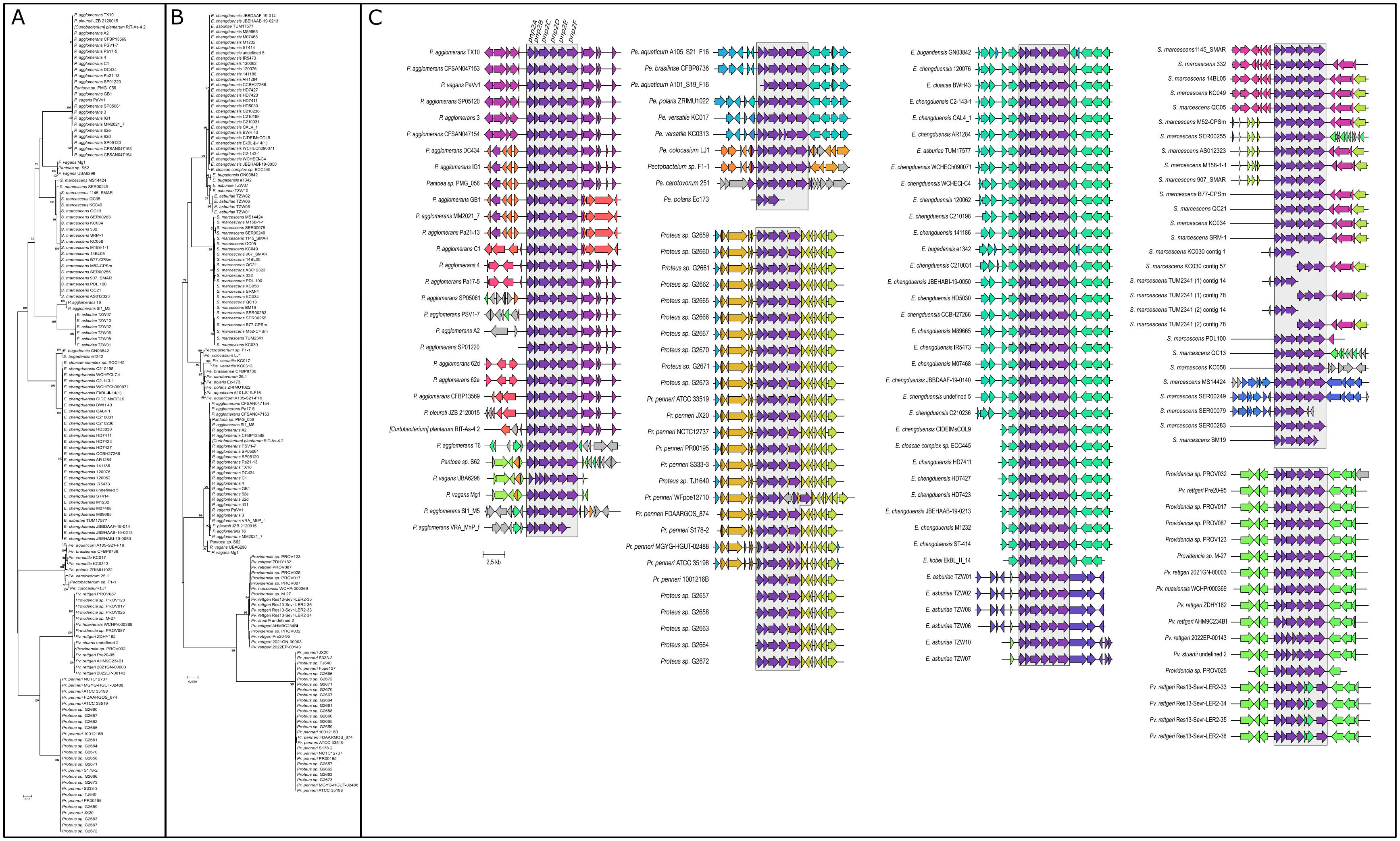


**Supplementary Figure 9.** Phylogenetic and comparative genomic analysis of *Pantoea* natural product 2 (PNP-2) clusters identified across bacterial strains. Panel A shows the maximum likelihood tree of PNP-2 clusters from representative strains, while panel B shows the maximum likelihood *cpn60* tree of strains carrying the PNP-2 cluster. Both trees were constructed using 1000 bootstrap replicates. Panel C shows the ORFs of each cluster (shown enclosed in the grey box) along with the genes flanking each end as predicted using GeneMark.hmm with Heuristic Models (Besemer and Borodovsky, 1999). Gene maps were generated with Clinker (Gilchrist and Chooi, 2021).

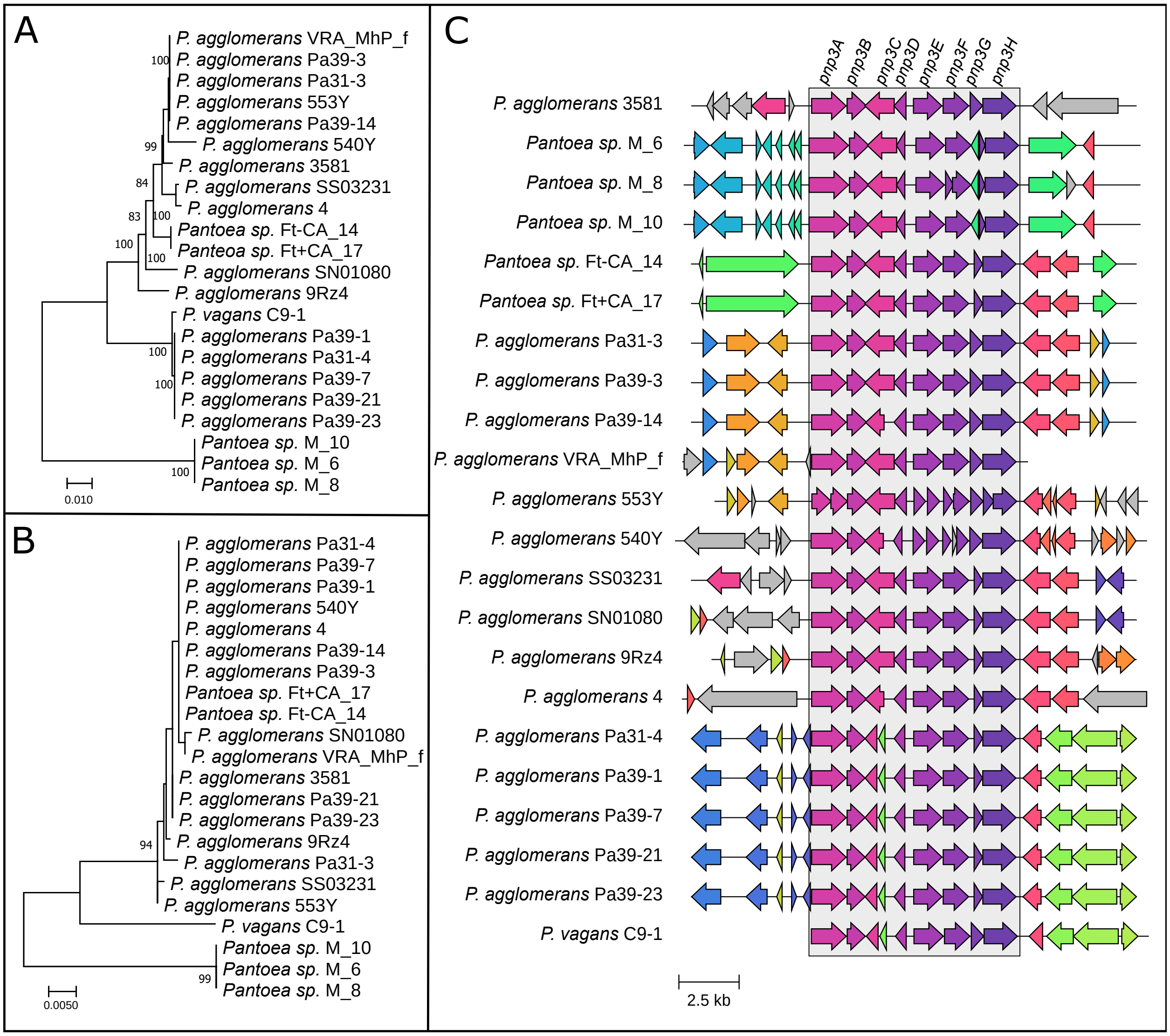


**Supplementary Figure 10.** Phylogenetic and comparative genomic analysis of *Pantoea* natural product 3 (PNP-3) clusters identified across bacterial strains. Panel A shows the maximum likelihood tree of PNP-3 clusters from representative strains, while panel B shows the maximum likelihood *cpn60* tree of strains carrying the PNP-3 cluster. Both trees were constructed using 1000 bootstrap replicates. Panel C shows the ORFs of each cluster (shown enclosed in the grey box) along with the genes flanking each end as predicted using GeneMark.hmm with Heuristic Models (Besemer and Borodovsky, 1999). Gene maps were generated with Clinker (Gilchrist and Chooi, 2021).

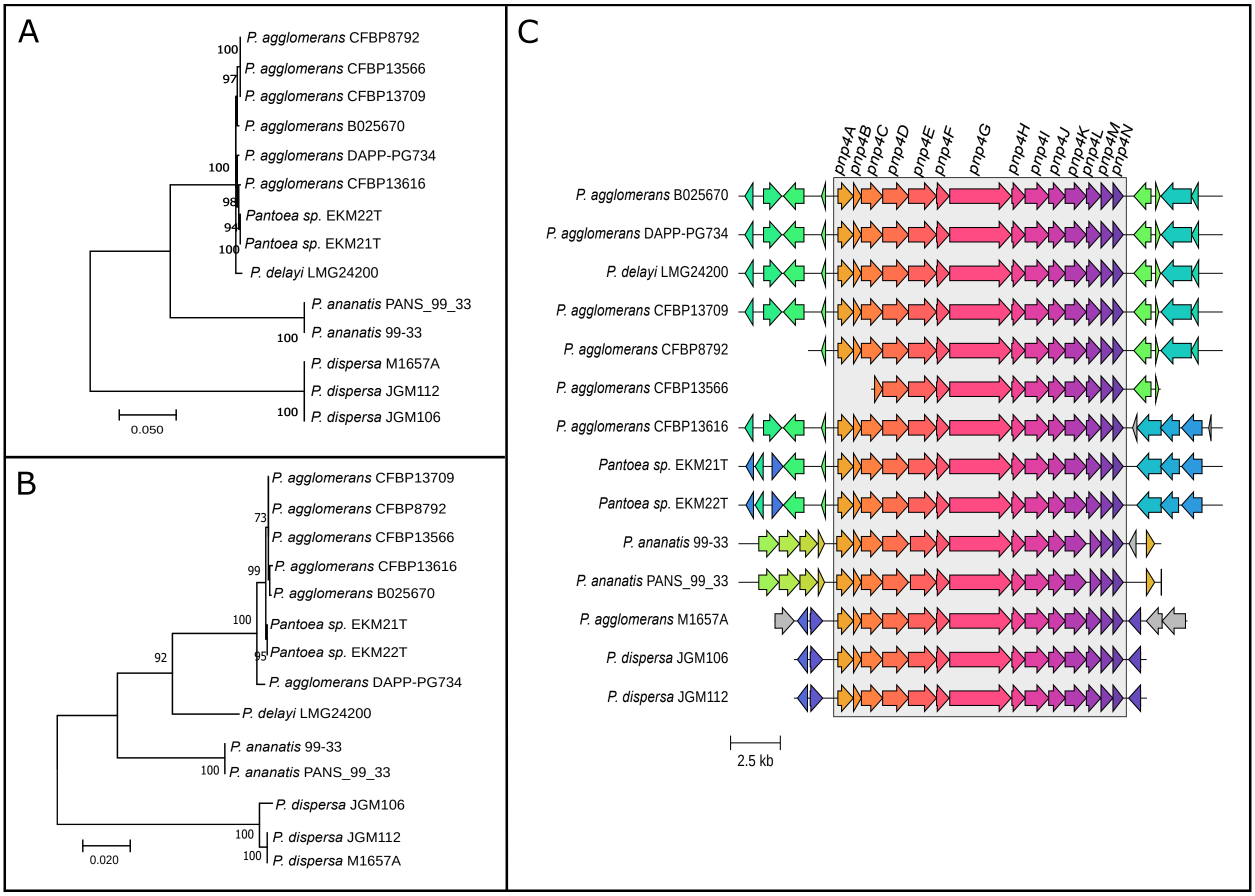


**Supplementary Figure 11.** Phylogenetic and comparative genomic analysis of *Pantoea* natural product 4 (PNP-4) clusters identified across bacterial strains. Panel A shows the maximum likelihood tree of PNP-4 clusters from representative strains, while panel B shows the maximum likelihood *cpn60* tree of strains carrying the PNP-4 cluster. Both trees were constructed using 1000 bootstrap replicates. Panel C shows the ORFs of each cluster (shown enclosed in the grey box) along with the genes flanking each end as predicted using GeneMark.hmm with Heuristic Models (Besemer and Borodovsky, 1999). Gene maps were generated with Clinker (Gilchrist and Chooi, 2021).

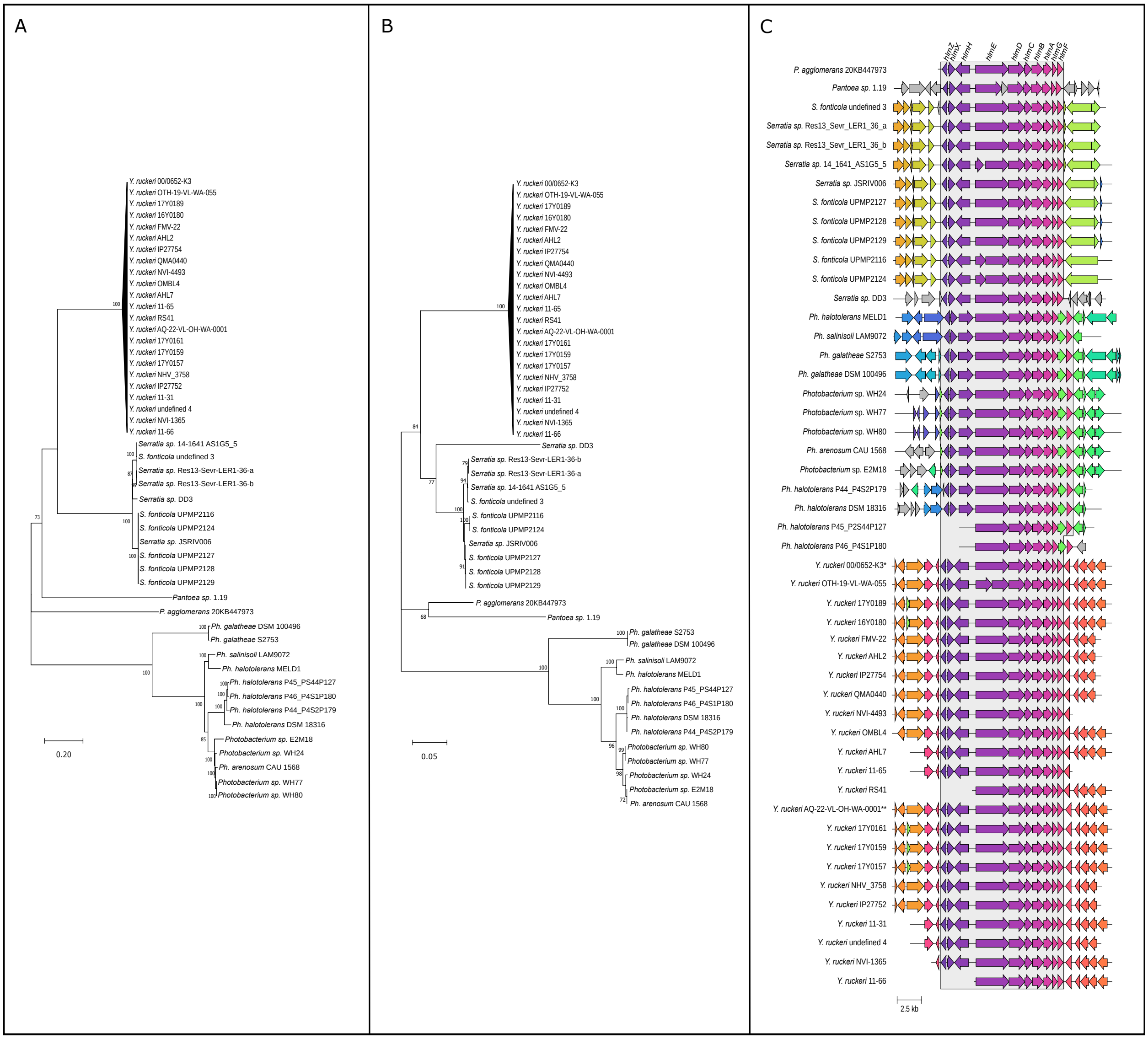


**Supplementary Figure 12.** Phylogenetic and comparative genomic analysis of *Pantoea* natural product 5 (PNP-5) clusters identified across bacterial strains. Panel A shows the maximum likelihood tree of PNP-5 clusters from representative strains, while panel B shows the maximum likelihood *cpn60* tree of strains carrying the PNP-5 cluster. Both trees were constructed using 1000 bootstrap replicates. Panel C shows the ORFs of each cluster (shown enclosed in the grey box) along with the genes flanking each end as predicted using GeneMark.hmm with Heuristic Models (Besemer and Borodovsky, 1999). Gene maps were generated with Clinker (Gilchrist and Chooi, 2021). The *Yersinia* clade has been condensed, with only some representatives being shown. Unique indels create two *Yersinia* gene cluster groups (indicated by * and **). The distribution of each of these across *Yersinia* strains is shown in Supplementary Table 5.

**Supplementary Table 1.** Number of representatives of queried genera in each database at NCBI at the time of our survey [accessed on October 10, 2023].

| Genus/Family | WGS Projects | nr Assemblies | Total |
|---|---|---|---|
| *Candidatus* Fukastuia | 0 | 2 | 2 |
| *Dickeya* | 309 | 207 | 516 |
| *Edwardsiella* | 119 | 122 | 241 |
| *Enterobacter* | 14,590 | 8,835 | 23,425 |
| *Erwinia* | 2,435 | 382 | 2,817 |
| *Musicola* | 2 | 4 | 6 |
| *Pantoea* | 1,641 | 886 | 2,527 |
| *Pectobacterium* | 744 | 438 | 1,182 |
| *Photobacterium* | 617 | 358 | 975 |
| *Proteus* | 3,061 | 1,858 | 4,919 |
| *Providencia* | 1,466 | 807 | 2,273 |
| *Serratia* | 4,292 | 2,641 | 6,933 |
| *Vibrio* | 35,990 | 20,827 | 56,817 |
| *Xenorhabdus* | 232 | 122 | 354 |
| *Yersinia* | 8,950 | 3,280 | 12,230 |

**Supplementary Table 2.** Models used for phylogenetic tree construction of *Pantoea* antibiotic gene clusters and *cpn60* sequences.*

| Cluster | Cluster Model | *cpn60* Model |
|---|---|---|
| Andrimid | GTR + G | K2 + G + I |
| D-alanylgriseoluteic acid | GTR + I | TN93 + G |
| Dapdiamide | K2 + G | TN93 + G |
| Herbicolin | N/A | N/A |
| Pantocin A | T92 + G | K2 + G + I |
| Pantocin B | HKY | TN93 |
| PNP-1 | T92 + I | TN93 |
| PNP-2 | GTR + G + I | K2 + G + I |
| PNP-3 | K2 + G + I | K2 + G |
| PNP-4 | GTR + G | TN93 + G |
| PNP-5 | GTR + G + I | TN93 + G + I |

***5 gamma categories used when applicable

**Supplementary Table 3.** Number of homologs of each antibiotic biosynthetic gene cluster found in each queried database.

| Cluster | Number of Representatives | | | |
|---|---|---|---|---|
| | nr | wgs | Strain Collection | Total |
| Agglomerin | 50 | 131 | 0 | 181 |
| Andrimid | 11 | 29 | 0 | 40 |
| AGA | 10 | 21 | 0 | 31 |
| Dapdiamide | 6 | 3 | 0 | 9 |
| Herbicolin | 2 | 1 | 0 | 4 |
| Pantocin A | 10 | 49 | 0 | 59 |
| Pantocin B | 0 | 2 | 0 | 3 |
| PNP-1 | 1 | 8 | 0 | 9 |
| PNP-2 | 21 | 123 | 4 | 148 |
| PNP-3 | 4 | 18 | 0 | 22 |
| PNP-4 | 3 | 10 | 1 | 14 |
| PNP-5 | 39 | 157 | 0 | 197 |

**Supplementary Table 4.** Distribution of *Pantoea* antibiotic biosynthetic gene clusters.

| Cluster | Database | Species | Strain | Query Cover | Percent Identity | E-Value | Accession Number |
|---|---|---|---|---|---|---|---|
| **Agglomerin** | **nr** | *Pantoea agglomerans* | PB-6042 | 100% | 100 | 0 | HF565364.1 |
| | | *Dickeya solani* | CFBP5647 | 97% | 88.59 | 0 | CP136339.1 |
| | | *Dickeya solani* | RNS 05.1.2A | 97% | 88.49 | 0 | CP104920.1 |
| | | *Dickeya solani* | IFB0421 | 97% | 88.29 | 0 | CP051460.1 |
| | | *Dickeya solani* | IFB0231 | 97% | 88.29 | 0 | CP051458.1 |
| | | *Dickeya solani* | IFB0167 | 97% | 88.29 | 0 | CP051457.1 |
| | | *Dickeya solani* | IFB0223 | 97% | 88.29 | 0 | CP024710.1 |
| | | *Dickeya solani* | IFB 0099 | 97% | 88.29 | 0 | CP024711.1 |
| | | *Dickeya solani* | PPO 9019 | 97% | 88.29 | 0 | CP017454.1 |
| | | *Dickeya solani* | D s0432-1 | 97% | 88.29 | 0 | CP017453.1 |
| | | *Dickeya solani* | RNS 08.23.3.1.A | 97% | 88.29 | 0 | CP016928.1 |
| | | *Dickeya solani* | IPO 2222 | 97% | 88.29 | 0 | CP015137.1 |
| | | *Dickeya solani* | DsR34 | 97% | 88.29 | 0 | CP110886.2 |
| | | *Dickeya solani* | DsR207 | 97% | 88.29 | 0 | CP110887.2 |
| | | *Dickeya solani* | IPO2019 | 97% | 88.29 | 0 | CP071062.1 |
| | | *Dickeya solani* | IFB0417 | 97% | 88.29 | 0 | CP051459.1 |
| | | *Dickeya dianthicola* | ME23 | 95% | 88.71 | 0 | CP031560.1 |
| | | *Dickeya dianthicola* | LAR.16.03.LID | 95% | 88.71 | 0 | CP038499.1 |
| | | *Dickeya dianthicola* | DDI_ME30 | 95% | 88.71 | 0 | CP076042.1 |
| | | *Dickeya dianthicola* | 16JP03 | 95% | 88.71 | 0 | CP069605.1 |
| | | *Dickeya dianthicola* | 16JP05 | 95% | 88.71 | 0 | CP069604.1 |
| | | *Dickeya dianthicola* | 16LI01 | 95% | 88.71 | 0 | CP069603.1 |
| | | *Dickeya dianthicola* | 16LI02 | 95% | 88.71 | 0 | CP069602.1 |
| | | *Dickeya dianthicola* | 16LI04 | 95% | 88.71 | 0 | CP069601.1 |
| | | *Dickeya dianthicola* | 16MA15T | 95% | 88.71 | 0 | CP069600.1 |

| | | | | | | |
|---|---|---|---|---|---|---|
| | *Dickeya dianthicola* | 16MB01 | 95% | 88.71 | 0 | CP069599.1 |
| | *Dickeya dianthicola* | 16ME21T | 95% | 88.71 | 0 | CP069598.1 |
| | *Dickeya dianthicola* | 16ME22T | 95% | 88.71 | 0 | CP069597.1 |
| | *Dickeya dianthicola* | 16SBJ16 | 95% | 88.71 | 0 | CP069596.1 |
| | *Dickeya dianthicola* | PA24 | 95% | 88.71 | 0 | CP069595.1 |
| | *Dickeya dadantii* | XJ12 | 97% | 88.5 | 0 | CP110362.1 |
| | *Dickeya dadantii* | 3937 | 97% | 88.51 | 0 | CP002038.1 |
| | *Dickeya dianthicola* | RNS04.9 | 95% | 88.69 | 0 | CP017638.1 |
| | *Dickeya sp.* | Secpp 1600 | 94% | 89.23 | 0 | CP023484.1 |
| | *Dickeya fangzhongdai* | PL145 | 94% | 89.23 | 0 | CP094338.1 |
| | *Dickeya dadantii* | FZ06 | 97% | 88.4 | 0 | CP094943.1 |
| | *Dickeya fangzhongdai* | DSM 101947 | 94% | 89.17 | 0 | CP025003.1 |
| | *Dickeya dianthicola* | 67-19 | 95% | 88.61 | 0 | CP051429.1 |
| | *Dickeya fangzhongdai* | QZH3 | 94% | 89.17 | 0 | CP031507.1 |
| | *Dickeya fangzhongdai* | LN1 | 94% | 89.17 | 0 | CP031505.1 |
| | *Dickeya dadantii* | DSM 18020 | 97% | 88.4 | 0 | CP023467.1 |
| | *Dickeya fangzhongdai* | PA1 | 94% | 89.12 | 0 | CP020872.1 |
| | *Dickeya dadantii* | S3-1 | 97% | 88.32 | 0 | CP076386.1 |
| | *Dickeya fangzhongdai* | Onc5 | 94% | 89.04 | 0 | CP080400.1 |
| | *Dickeya fangzhongdai* | B16 | 94% | 89.04 | 0 | CP087226.1 |
| | *Dickeya fangzhongdai* | ZXC1 | 94% | 89.02 | 0 | CP119773.1 |
| | *Dickeya dadantii* | M2-3 | 97% | 88.25 | 0 | CP077422.1 |
| | *Dickeya fangzhongdai* | 643b | 94% | 89.12 | 0 | CP092458.1 |
| | *Dickeya fangzhongdai* | AP6 | 94% | 88.94 | 0 | CP092460.1 |
| | *Dickeya fangzhongdai* | ND14b | 95% | 88.95 | 0 | CP009460.1 |
| **wgs** | *Dickeya chrysanthemi* | EC16 | 100% | 98.78 | 0 | JAFCAF010000003.1 |
| | *Dickeya chrysanthemi* | NCPPB 402 | 100% | 98.78 | 0 | AOOA01000022.1 |
| | *Dickeya chrysanthemi* | A604-S21-A17 | 100% | 98.51 | 0 | JAIZFX010000004.1 |
| | *Dickeya solani* | FVG2-MFV017-A9 | 97% | 88.49 | 0 | JAWLLM010000001.1 |

| | | | | | |
|---|---|---|---|---|---|
| *Dickeya solani* | FVG9-S3-A17-E1 | 97% | 88.49 | 0 | JAWLLL010000007.1 |
| *Dickeya solani* | FVG13-S21A17-D9 | 97% | 88.49 | 0 | JAWLLK010000010.1 |
| *Dickeya solani* | FVG14-S21A17-C8 | 97% | 88.49 | 0 | JAWLLJ010000023.1 |
| *Dickeya solani* | RNS10-105-1A | 97% | 88.49 | 0 | JAPTND010000019.1 |
| *Dickeya solani* | A623-S20-A17 | 97% | 88.49 | 0 | JAIZGA010000001.1 |
| *Dickeya solani* | M21a | 97% | 88.29 | 0 | VZQM01000018.1 |
| *Dickeya solani* | Sp1a | 97% | 88.29 | 0 | VZQL01000022.1 |
| *Dickeya solani* | Am3a | 97% | 88.29 | 0 | VZQK01000001.1 |
| *Dickeya solani* | RNS10-27-2A | 97% | 88.29 | 0 | VZQJ01000009.1 |
| *Dickeya solani* | MIE35 | 97% | 88.29 | 0 | VZQI01000015.1 |
| *Dickeya solani* | D12 | 97% | 88.29 | 0 | PGUT01000005.1 |
| *Dickeya solani* | F012 FGLHLNJM_2 | 97% | 88.29 | 0 | PGOJ01000002.1 |
| *Dickeya solani* | IFB_0158 | 97% | 88.29 | 0 | PENA01000002.1 |
| *Dickeya solani* | IFB_0221 | 97% | 88.29 | 0 | PEMZ01000002.1 |
| *Dickeya solani* | F012 | 97% | 88.29 | 0 | PDVN01000002.1 |
| *Dickeya solani* | PPO 9134 | 97% | 88.29 | 0 | JWLT01000004.1 |
| *Dickeya solani* | PPO 9019 | 97% | 88.29 | 0 | JWLS01000001.1 |
| *Dickeya solani* | RNS 07.7.3B | 97% | 88.29 | 0 | JWLR01000011.1 |
| *Dickeya solani* | PO3796 | 97% | 88.29 | 0 | JAWLLI010000005.1 |
| *Dickeya solani* | RNS13-0-1B | 97% | 88.29 | 0 | JAPTNC010000001.1 |
| *Dickeya solani* | RNS13-31-1A | 97% | 88.29 | 0 | JAPTNB010000032.1 |
| *Dickeya solani* | RNS13-48-1A | 97% | 88.29 | 0 | JAPTNA010000013.1 |
| *Dickeya solani* | RNS15-102-1A | 97% | 88.29 | 0 | JAPTMZ010000035.1 |
| *Dickeya solani* | CH07044 | 97% | 88.29 | 0 | JAEKIP010000002.1 |
| *Dickeya solani* | CH05026 | 97% | 88.29 | 0 | JAEKIO010000001.1 |
| *Dickeya solani* | CH9918-774 | 97% | 88.29 | 0 | JAEKIN010000007.1 |
| *Dickeya solani* | CH9635-1 | 97% | 88.29 | 0 | JAEKIM010000009.1 |
| *Dickeya solani* | IFB0695 | 97% | 88.29 | 0 | JABAOQ010000003.1 |
| *Dickeya solani* | IFB0487 | 97% | 88.29 | 0 | JABAOP010000002.1 |

| | | | | | |
|---|---|---|---|---|---|
| *Dickeya solani* | IFB0311 | 97% | 88.29 | 0 | JABAOO010000001.1 |
| *Dickeya solani* | IFB0212 | 97% | 88.29 | 0 | JABAON010000001.1 |
| *Dickeya solani* | MK16 | 97% | 88.29 | 0 | AOOQ01000006.1 |
| *Dickeya solani* | GBBC 2040 | 90% | 88.29 | 0 | AONX01000115.1 |
| *Dickeya solani* | IPO 2222 | 90% | 88.29 | 0 | AONU01000039.1 |
| *Dickeya solani* | D s0432-1 | 97% | 88.29 | 0 | AMWE01000002.1 |
| *Dickeya solani* | MK10 | 97% | 88.27 | 0 | AOOP01000010.1 |
| *Dickeya sp.* | NCPPB 3274 | 97% | 87.87 | 0 | AOOH01000021.1 |
| *Dickeya dadantii* subsp. *dieffenbachiae* | NCPPB 2976 | 95% | 88.9 | 0 | AOOG01000028.1 |
| *Dickeya dianthicola* | CFBP6548 | 94% | 88.97 | 0 | JALDOI010000010.1 |
| *Dickeya dianthicola* | CH90110-7-1 | 94% | 88.97 | 0 | JALDOF010000003.1 |
| *Dickeya dianthicola* | IPO256 | 93% | 88.97 | 0 | JALDOD010000015.1 |
| *Dickeya dianthicola* | IPO1003 | 94% | 88.97 | 0 | JALDNY010000072.1 |
| *Dickeya dianthicola* | IPO1350 | 93% | 88.97 | 0 | JALDNW010000129.1 |
| *Dickeya dianthicola* | IPO3846 | 94% | 88.97 | 0 | JALDNP010000004.1 |
| *Dickeya dianthicola* | GBBC 2039 | 82% | 88.97 | 0 | AOOM01000087.1 |
| *Dickeya dianthicola* | SS70 | 95% | 88.92 | 0 | QESZ01000012.1 |
| *Dickeya dianthicola* | IPO3797 | 93% | 88.92 | 0 | JALDNR010000037.1 |
| *Dickeya dianthicola* | CFBP3706 | 94% | 88.89 | 0 | JALDOJ010000045.1 |
| *Dickeya dianthicola* | CH88.23 | 95% | 88.89 | 0 | JALDOH010000005.1 |
| *Dickeya dianthicola* | CH9187-1 | 94% | 88.89 | 0 | JALDOE010000029.1 |
| *Dickeya dianthicola* | NY1785A | 93% | 88.71 | 0 | WABK01000054.1 |
| *Dickeya dianthicola* | NY1760A | 93% | 88.71 | 0 | WABJ01000054.1 |
| *Dickeya dianthicola* | NY1758A | 93% | 88.71 | 0 | WABI01000053.1 |
| *Dickeya dianthicola* | NY1746A | 93% | 88.71 | 0 | WABH01000055.1 |
| *Dickeya dianthicola* | NY1719A | 93% | 88.71 | 0 | WABG01000085.1 |
| *Dickeya dianthicola* | NY1713C | 93% | 88.71 | 0 | WABF01000079.1 |
| *Dickeya dianthicola* | NY1578A | 93% | 88.71 | 0 | WABE01000050.1 |

| | | | | | |
|---|---|---|---|---|---|
| *Dickeya dianthicola* | NY1562C | 93% | 88.71 | 0 | WABD01000046.1 |
| *Dickeya dianthicola* | NY1559C | 93% | 88.71 | 0 | WABC01000050.1 |
| *Dickeya dianthicola* | NY1558D | 93% | 88.71 | 0 | WABB01000058.1 |
| *Dickeya dianthicola* | NY1557A | 93% | 88.71 | 0 | WABA01000082.1 |
| *Dickeya dianthicola* | NY1556C | 93% | 88.71 | 0 | WAAZ01000054.1 |
| *Dickeya dianthicola* | NY1547B | 93% | 88.71 | 0 | WAAY01000036.1 |
| *Dickeya dianthicola* | NY1538B | 93% | 88.71 | 0 | WAAX01000056.1 |
| *Dickeya dianthicola* | NY1536B | 93% | 88.71 | 0 | WAAW01000051.1 |
| *Dickeya dianthicola* | NY1528B | 93% | 88.71 | 0 | WAAV01000060.1 |
| *Dickeya dianthicola* | MIE34 | 93% | 88.71 | 0 | VZQH01000059.1 |
| *Dickeya dianthicola* | CFBP2015 | 93% | 88.71 | 0 | VZQG01000005.1 |
| *Dickeya dianthicola* | CFBP2982 | 94% | 88.71 | 0 | VZQF01000043.1 |
| *Dickeya dianthicola* | RNS 11-47-1-1A | 93% | 88.71 | 0 | VYSC01000047.1 |
| *Dickeya dianthicola* | S4.16.03.LID | 93% | 88.71 | 0 | QZDO01000044.1 |
| *Dickeya dianthicola* | S4.16.03.P2.4 | 93% | 88.71 | 0 | QZDN01000070.1 |
| *Dickeya dianthicola* | WV516 | 93% | 88.71 | 0 | PJJC01000029.1 |
| *Dickeya dianthicola* | DE440 | 93% | 88.71 | 0 | PJJB01000017.1 |
| *Dickeya dianthicola* | CFBP1805 | 94% | 88.71 | 0 | JALDOM010000003.1 |
| *Dickeya dianthicola* | CFBP1984 | 93% | 88.71 | 0 | JALDOL010000030.1 |
| *Dickeya dianthicola* | CFBP2598 | 94% | 88.71 | 0 | JALDOK010000037.1 |
| *Dickeya dianthicola* | CH8885 | 93% | 88.71 | 0 | JALDOG010000009.1 |
| *Dickeya dianthicola* | IPO502 | 93% | 88.71 | 0 | JALDOC010000025.1 |
| *Dickeya dianthicola* | IPO846 | 93% | 88.71 | 0 | JALDOA010000073.1 |
| *Dickeya dianthicola* | IPO973 | 93% | 88.71 | 0 | JALDNZ010000052.1 |
| *Dickeya dianthicola* | IPO3646 | 93% | 88.71 | 0 | JALDNU010000074.1 |
| *Dickeya dianthicola* | IPO3699 | 93% | 88.71 | 0 | JALDNT010000052.1 |
| *Dickeya dianthicola* | IPO3700 | 93% | 88.71 | 0 | JALDNS010000022.1 |
| *Dickeya dianthicola* | IPO3845 | 93% | 88.71 | 0 | JALDNQ010000023.1 |
| *Dickeya dianthicola* | IPO 980 | 95% | 88.71 | 0 | AOOS01000023.1 |

| | | | | | |
|---|---|---|---|---|---|
| *Dickeya dianthicola* | NCPPB 3534 | 93% | 88.71 | 0 | AOOK01000018.1 |
| *Dickeya dianthicola* | CFBP1888 | 93% | 88.69 | 0 | VZQE01000040.1 |
| *Dickeya dianthicola* | IPO1741 | 94% | 88.69 | 0 | JALDNV010000035.1 |
| *Dickeya dianthicola* | A260-S21-A16 | 94% | 88.69 | 0 | JAIZFZ010000020.1 |
| *Dickeya dianthicola* | DDI_16NJ12 | 95% | 88.69 | 0 | JAHEPV010000004.1 |
| *Dickeya dianthicola* | RNS04.9 | 95% | 88.69 | 0 | APVF01000002.1 |
| *Dickeya dianthicola* | NCPPB 453 | 94% | 88.69 | 0 | AOOB01000016.1 |
| *Dickeya dadantii* | NCPPB 898 | 97% | 88.45 | 0 | AOOE01000019.1 |
| *Dickeya dadantii* | CZ1501 | 95% | 88.46 | 0 | MPDL01000255.1 |
| *Dickeya fangzhongdai* | CGMCC 1.15464 | 94% | 89.17 | 0 | BMJF01000001.1 |
| *Dickeya dadantii* | NCPPB 3537 | 97% | 88.42 | 0 | AOOL01000016.1 |
| *Dickeya dadantii* | Kunimi-3 | 95% | 88.41 | 0 | SMHE01000019.1 |
| *Dickeya dadantii* | BI3-1 | 95% | 88.41 | 0 | PHRA01000042.1 |
| *Dickeya dadantii* | Yana2-2 | 95% | 88.41 | 0 | JABEPB010000034.1 |
| *Dickeya dadantii* | Aka1-1 | 81% | 88.41 | 0 | JABEPA010000069.1 |
| *Dickeya dadantii* | BI1-1 | 97% | 88.38 | 0 | JABEOZ010000041.1 |
| *Dickeya dadantii* | Housui2-1 | 95% | 88.22 | 0 | JABEPD010000035.1 |
| *Dickeya dadantii* | Kousui1-1 | 75% | 88.22 | 0 | JABEPC010000101.1 |
| *Dickeya sp.* | MK7 | 94% | 89.02 | 0 | AOOO01000021.1 |
| *Dickeya dianthicola* | DDI_59W | 95% | 88.43 | 0 | JAHEPW010000004.1 |
| *Dickeya dianthicola* | DDI_16NJ11 | 95% | 88.43 | 0 | JAHEPU010000006.1 |
| *Dickeya dianthicola* | DDI_16PA07 | 95% | 88.43 | 0 | JAHEPT010000003.1 |
| *Dickeya dadantii* | ICMP 9290 | 97% | 88.19 | 0 | SGPP01000009.1 |
| *Dickeya fangzhongdai* | S1 | 94% | 88.96 | 0 | JXBO02000002.1 |
| *Dickeya fangzhongdai* | 908C | 94% | 88.96 | 0 | JADCNJ010000029.1 |
| *Dickeya dadantii* | A622-S1-A17 | 95% | 88.31 | 0 | JAIZFY010000021.1 |
| *Dickeya fangzhongdai* | M005 | 95% | 88.98 | 0 | JSXD01000024.1 |
| *Dickeya fangzhongdai* | M074 | 95% | 88.95 | 0 | JRWY01000098.1 |
| *Musicola paradisiaca* | NCPPB 2511 | 92% | 83.54 | 0 | AONV01000025.1 |

| | | | | | | | |
|---|---|---|---|---|---|---|---|
| | | *Dickeya oryzae* | S20 | 92% | 83.39 | 0 | JAGJWV010000015.1 |
| | | *Dickeya oryzae* | A642-S2-A17 | 92% | 83.28 | 0 | JAIZGC010000002.1 |
| | | *Dickeya oryzae* | A003-S1-M15 | 92% | 83.25 | 0 | JAIZGB010000015.1 |
| | | *Dickeya oryzae* | FVG03 | 92% | 83.23 | 0 | JAGJWX010000011.1 |
| | | *Dickeya parazeae* | S31 | 92% | 83.25 | 0 | JAGJWU010000011.1 |
| | | *Dickeya zeae* | NCPPB 3531 | 94% | 83.18 | 0 | AOOI01000013.1 |
| | | *Dickeya oryzae* | CSL RW192 | 93% | 83.18 | 0 | AONY01000021.1 |
| | | *Dickeya oryzae* | BRIP64262 | 92% | 83.18 | 0 | JAMXSN010000012.1 |
| | | *Musicola keenii* | A3967 | 92% | 83 | 0 | JAAWVW010000002.1 |
| | | *Dickeya dianthicola* | IPO1348 | 52% | 88.05 | 0 | JALDNX010000176.1 |
| | | *Dickeya dianthicola* | IPO775 | 52% | 88.01 | 0 | JALDOB010000132.1 |
| | **collection** | N/A | | | | | |
| **Andrimid** | **nr** | *Pantoea agglomerans* | Eh355 | 100% | 100% | 0 | AY192157.1 |
| | | *Serratia plymuthica* | UBCF_13 | 97% | 87.32 | 0 | CP068771.1 |
| | | *Serratia plymuthica* | NCTC8015 | 98% | 87.3 | 0 | LR134478.1 |
| | | *Serratia plymuthica* | NCTC8900 | 98% | 87.29 | 0 | LR134151.1 |
| | | *Serratia marcescens* | LVF3 | 96% | 83.22 | 0 | CP063229.1 |
| | | *Serratia marcescens* | E05 | 96% | 83.35 | 0 | AP028491.1 |
| | | *Serratia marcescens* | E38 | 96% | 83.33 | 0 | AP028510.1 |
| | | *Serratia ureilytica* | HNU47 | 96% | 83.48 | 0 | CP098030.1 |
| | | *Serratia plymuthica* | C-1 | 97% | 85.43 | 0 | CP053398.1 |
| | | *Vibrio coralliilyticus* | OCN008 | 92% | 73.94 | 0 | CP048695.1 |
| | | *Vibrio coralliilyticus* | S2052 | 92% | 73.94 | 0 | CP063053.1 |
| | **wgs** | *Pantoea sp.* | B623 | 99% | 91.16 | 0 | JANUXM010000004.1 |
| | | *Pantoea ananatis* | 99-26 | 99% | 91.16 | 0 | JALKJM010000001.1 |
| | | *Pantoea stewartii* subsp. *Indologenes* | PANS 07-6 | 99% | 91.16 | 0 | JADWWG010000004.1 |
| | | *Pantoea ananatis* | PANS_99_26 | 99% | 91.16 | 0 | JABDZI010000001.1 |
| | | *Pantoea ananatis* | PANS 200-1 | 99% | 91.16 | 0 | QTTV01000001.1 |

| | | | | | | |
|---|---|---|---|---|---|---|
| | *Pantoea sp.* | B566 | 99% | 91.16 | 0 | JANUQL010000003.1 |
| | *Pantoea ananatis* | 200-1 | 99% | 91.16 | 0 | JALKLB010000001.1 |
| | *Pantoea stewartii* | PANS 07-4 | 99% | 91.16 | 0 | JADWWF010000005.1 |
| | *Pantoea ananatis* | PANS_200_1 | 99% | 91.16 | 0 | JABDZB010000001.1 |
| | *Pantoea ananatis* | BAV 3296 | 99% | 91.15 | 0 | WHOW01000051.1 |
| | *Serratia proteamaculans* | 72f938ee-a76c-11e8-a962-3c4a9275d6c8 | 97% | 87.74 | 0 | CAMKPQ010000003.1 |
| | *Serratia proteamaculans* | 74cc738e-a76c-11e8-a962-3c4a9275d6c | 97% | 87.74 | 0 | CAMITN010000001.1 |
| | *Serratia proteamaculans* | 6e3de084-a76c-11e8-a962-3c4a9275d6c8 | 97% | 87.7 | 0 | CAMISV010000007.1 |
| | *Serratia marcescens* | MSU97 | 97% | 84.61% | 0 | MJAO01000010.1 |
| | *Serratia marcescens* | KS23 | 96% | 83.31 | 0 | RCDR01000134.1 |
| | *Serratia marcescens* | ID149856 | 96% | 83.3 | 0 | PQOH01000001.1 |
| | *Serratia marcescens* | ID149855 | 96% | 83.3 | 0 | PQOG01000001.1 |
| | *Serratia marcescens* | ID148696 | 96% | 83.3 | 0 | PQOB01000001.1 |
| | *Serratia marcescens* | SER00097 | 96% | 83.24 | 0 | JADTUT010000001.1 |
| | *Serratia marcescens* | MGH136 | 96% | 83.23 | 0 | NGUE01000001.1 |
| | *Serratia marcescens* | SER00116 | 96% | 83.22 | 0 | JADTUM010000001.1 |
| | *Serratia marcescens* | 90-166 | 96% | 83.48 | 0 | LCWI01000001.1 |
| | *Serratia ureilytica* | HEE16_1 | 96% | 83.48 | 0 | JASBWN010000001.1 |
| | *Serratia sp.* | S1C92_SP369 | 96% | 83.47 | 0 | CAJYDV010000019.1 |
| | *Serratia sp.* | X10 | 96% | 83.46 | 0 | JAEIOH010000002.1 |
| | *Serratia sp.* | X3 | 96% | 83.46 | 0 | JADCNB010000001.1 |
| | *Serratia plymuthica* | A153 | 97% | 87.14 | 0 | LRQU01000001.1 |
| | *Serratia marcescens* | E29 | 84% | 83.96 | 0 | BPZB01000009.1 |
| | *Serratia plymuthica* | undefined 1 | 96% | 85.45 | 0 | CAQO01000014.1 |
| **collection** | N/A | | | | | |
| **nr** | *Pantoea agglomerans* | Eh1087 | 100% | 100% | 0 | AF451953.1 |

| | | | | | | | |
|---|---|---|---|---|---|---|---|
| **D-alanylgriseoluteic acid (AGA)** | | *Pantoea agglomerans* | ASB05 | 100% | 99.67 | 0 | CP046724.1 |
| | | *Pantoea agglomerans* | CFSAN047153 | 100% | 99.67 | 0 | CP034471.1 |
| | | *Pantoea agglomerans* | CFSAN047154 | 100% | 99.67 | 0 | CP034476.1 |
| | | *Pectobacterium sp.* | F1-1 | 99% | 86.57 | 0 | CP104733.1 |
| | | *Pectobacterium parvum* | FN20211 | 99% | 86.8 | 0 | CP087392.1 |
| | | *Pectobacterium parvum* | YT22221 | 99% | 86.8 | 0 | CP102749.1 |
| | | *Xenorhabdus nematophila* | YL001 | 72% | 65.74 | 5.00E-157 | CP032329.1 |
| | | *Xenorhabdus nematophila* | SII | 72% | 65.74 | 5.00E-157 | CP060401.1 |
| | | *Xenorhabdus sp.* | SF857 | 73% | 66.94 | 0 | CP119194.1 |
| | **wgs** | *Pantoea agglomerans* | ICMP 1087 | 100% | 99.8 | 0 | SGQI01000008.1 |
| | | *Pantoea agglomerans* | BAV 2934 | 100% | 99.67 | 0 | WHOZ01000004.1 |
| | | *Pantoea agglomerans* | 190 | 100% | 99.67 | 0 | JNGC01000004.1 |
| | | *Pantoea agglomerans* | Pa39-21 | 100% | 99.67 | 0 | JACSWX010000022.1 |
| | | *Pantoea agglomerans* | Pa39-23 | 100% | 99.67 | 0 | JACSWW010000013.1 |
| | | *Pantoea agglomerans* | Pa39-7 | 100% | 99.64 | 0 | JACSWZ010000008.1 |
| | | *Xenorhabdus bovienii* | MC_239_E_2016 | 79% | 64.92 | 0 | JAILSS010000038.1 |
| | | *Xenorhabdus bovienii* | MC_59 | 79% | 64.91 | 0 | JAILTL010000078.1 |
| | | *Xenorhabdus bovienii* | MC_67 | 79% | 64.91 | 0 | JAILTJ010000078.1 |
| | | *Xenorhabdus bovienii* | MC_266_E_2016 | 79% | 64.91 | 0 | JAILSO010000071.1 |
| | | *Xenorhabdus nematophila* | SII-2019 | 72% | 65.74 | 2.00E-160 | WUUN01000064.1 |
| | | *Xenorhabdus nematophila* | SC 0516 | 72% | 65.74 | 2.00E-160 | JACDOS010000001.1 |
| | | *Xenorhabdus nematophila* | Websteri | 72% | 65.74 | 2.00E-160 | CCWW01000105.1 |
| | | *Xenorhabdus nematophila* | C2-3 | 72% | 65.75 | 6.00E-160 | JRJV01000041.1 |
| | | *Pectobacterium parvum* | IFB5220 | 99% | 86.82 | 0 | PHSZ01000040.1 |
| | | *Pectobacterium polaris* | s0421 | 99% | 86.82 | 0 | OANP03000017.1 |
| | | *Pectobacterium polaris* | s0416 | 99% | 86.82 | 0 | OANO03000035.1 |
| | | *Pectobacterium parvum* | NCPPB 3395 | 99% | 86.82 | 0 | JQHN01000043.1 |
| | | *Pectobacterium polaris* | WBC11 | 99% | 86.8 | 0 | WUBF01000044.1 |

| | | | | | | | |
|---|---|---|---|---|---|---|---|
| | | *Pectobacterium polaris* | WBC9 | 99% | 86.8 | 0 | WUBE01000044.1 |
| | | *Pectobacterium carotovorum* subsp. *carotovorum* | MAFF 301879 | 81% | 86.76 | 0 | BRCW01000048.1 |
| | **collection** | N/A | | | | | |
| **Dapdiamide** | **nr** | *Pantoea agglomerans* | CU0119 | 100% | 100 | 0 | HQ130277.1 |
| | | *Pantoea agglomerans* | 48b/90 | 100% | 99.64 | 0 | JQ901494.1 |
| | | *Pantoea agglomerans* | C410P1 | 99% | 98.08 | 0 | CP016891.1 |
| | | *Pantoea agglomerans* | DAPP-PG734 | 99% | 96.38 | 0 | OW970319.1 |
| | | *Pantoea vagans* | C9-1 | 100% | 95.6 | 0 | CP001894.1 |
| | | *Serratia inhibens* | PRI-2C | 99% | 81.24 | 0 | CP015613.1 |
| | **wgs** | *Pantoea agglomerans* | 4 | 100% | 99.98 | 0 | JPOT02000004.1 |
| | | *Pantoea agglomerans* | 540Y | 100% | 99.25 | 0 | JAIWZA010000006.1 |
| | | *Pantoea agglomerans* | Sl1_M5 | 99% | 98.18 | 0 | ADWZ01000022.1 |
| | **collection** | N/A | | | | | |
| **Herbicolin** | **nr** | *Pantoea agglomerans* | ZJU23 | 100% | 100 | 0 | CP068441.1 |
| | | *Pantoea sp.* | CCBC3-3-1 | 99% | 77.3 | 0 | CP034363.1 |
| | | *Candidatus* Fukatsuia symbiotica | 5D | 82% | 66.13 | 0 | CP021660.1 |
| | **wgs** | *Pantoea agglomerans* | 9Rz4 | 100% | 99.03 | 0 | JANHBZ010000004.1 |
| | **collection** | N/A | | | | | |
| **Pantocin A** | **nr** | *Pantoea agglomerans* | Eh318 | 100% | 100 | 0 | U81376.2 |
| | | *Pantoea agglomerans* | 3581r | 99% | 96.8 | 0 | MT711881.1 |
| | | *Pantoea vagans* | C9-1 | 100% | 96.69 | 0 | CP002206.1 |
| | | *Dickeya chrysanthemi* | Ech1591 | 98% | 83.2 | 0 | CP001655.1 |
| | | *Edwardsiella hoshinae* | FDAARGOS_940 | 99% | 74.64 | 0 | CP065626.1 |
| | | *Dickeya chrysanthemi* | BRR1 | 98% | 83.28 | 0 | CP128510.1 |
| | | *Pectobacteriaceae* bacterium | C52 | 95% | 71.64 | 0 | CP128861.1 |
| | | *Pectobacteriaceae* bacterium | C80 | 95% | 71.6 | 0 | CP129116.1 |
| | | *Pectobacteriaceae* bacterium | CE70 | 95% | 71.6 | 0 | CP128863.1 |

| | | | | | | |
|---|---|---|---|---|---|---|
| | *Pectobacteriaceae* bacterium | CE90 | 95% | 71.35 | 0 | CP129114.1 |
| **wgs** | *[Curtobacterium] plantarum* (*Pantoea agglomerans*) | LMG 16222 | 100% | 99.63 | 0 | RHDS01000006.1 |
| | *Pantoea agglomerans* | P10c | 100% | 99.37 | 0 | LIME01000008.1 |
| | *Pantoea agglomerans* | Pa31-3 | 100% | 98.66 | 0 | JACSXF010000021.1 |
| | *Pantoea brenneri* | LMG 5343 | 99% | 98.91 | 0 | MIEI01000127.1 |
| | *Pantoea agglomerans* | BD 1274 | 100% | 98.51 | 0 | QQXI01000204.1 |
| | *Pantoea agglomerans* | 20TX0122 | 100% | 98.51 | 0 | JAKZMS010000007.1 |
| | *Pantoea agglomerans* | Pa39-3 | 99% | 98.65 | 0 | JACSXB010000007.1 |
| | *Pantoea agglomerans* | 540Y | 99% | 98.58 | 0 | JAIWZA010000001.1 |
| | *Pantoea ananatis* | PNA 14-1 | 99% | 98.16 | 0 | QEKS01000026.1 |
| | *Pantoea ananatis* | 14-2 | 99% | 98.16 | 0 | JALKIX010000003.1 |
| | *Pantoea ananatis* | PNA_14_2 | 99% | 98.16 | 0 | JABEAH010000005.1 |
| | *Pantoea agglomerans* | Pa39-1 | 98% | 98.37 | 0 | JACSXC010000034.1 |
| | *Pantoea agglomerans* | Tx10 | 98% | 98.37 | 0 | ASJI01000014.1 |
| | *Pantoea agglomerans* | 553Y | 100% | 96.33 | 0 | JAIWYZ010000001.1 |
| | *Pantoea stewartii* | NRRL B-133 | 99% | 95.67 | 0 | JACETZ010000004.1 |
| | *Pantoea sp.* | ICBG 985 | 99% | 95.7 | 0 | POWM01000010.1 |
| | *Pantoea sp.* | Ap-870 | 99% | 95.43 | 0 | VWXG01000003.1 |
| | *Pantoea sp.* | Taur | 100% | 95.17 | 0 | SZZZ01000007.1 |
| | *Pantoea ananatis* | MMB-1 | 100% | 90.97 | 0 | JAABOW010000010.1 |
| | *Pantoea ananatis* | 99-27 | 99% | 90.82 | 0 | JALKLD010000004.1 |
| | *Pantoea ananatis* | 98-3 | 99% | 90.82 | 0 | JALKKW010000004.1 |
| | *Pantoea ananatis* | 01-1 | 99% | 90.82 | 0 | JALKJG010000015.1 |
| | *Pantoea ananatis* | 1-10 | 99% | 90.82 | 0 | JALKIS010000002.1 |
| | *Pantoea ananatis* | PNA_98_3 | 99% | 90.82 | 0 | JABEBI010000002.1 |
| | *Pantoea ananatis* | PANS_99_27 | 99% | 90.82 | 0 | JABDZJ010000002.1 |
| | *Pantoea ananatis* | PANS_1_9 | 99% | 90.82 | 0 | JABDYV010000001.1 |
| | *Pantoea ananatis* | PANS_1_10 | 99% | 90.82 | 0 | JABDYQ010000002.1 |

| | | | | | | | |
|---|---|---|---|---|---|---|---|
| | | *Pantoea ananatis* | PNA 15-1 | 99% | 90.79 | 0 | NMZZ01000005.1 |
| | | *Pantoea ananatis* | PNA 99-7 | 99% | 90.79 | 0 | NMZW01000001.1 |
| | | *Pantoea stewartii* | ST25 | 99% | 90.79 | 0 | JAOSLH010000009.1 |
| | | *Pantoea ananatis* | 99-5 | 99% | 90.79 | 0 | JALKLI010000005.1 |
| | | *Pantoea ananatis* | 15-1 | 99% | 90.79 | 0 | JALKKP010000001.1 |
| | | *Pantoea ananatis* | PNA_15_1 | 99% | 90.79 | 0 | JABEAI010000001.1 |
| | | *Pantoea ananatis* | PANS_99_5 | 99% | 90.79 | 0 | JABDZQ010000001.1 |
| | | *Pantoea ananatis* | 18-8S | 99% | 90.71 | 0 | JALKLA010000035.1 |
| | | *Pantoea ananatis* | 18-9S | 99% | 90.71 | 0 | JALKJV010000035.1 |
| | | *Pantoea ananatis* | 18-6S | 99% | 90.71 | 0 | JALKJA010000037.1 |
| | | *Pantoea ananatis* | 18-10S | 99% | 90.71 | 0 | JALKIZ010000036.1 |
| | | *Pantoea ananatis* | PNA_18_9S | 99% | 90.71 | 0 | JABEAU010000003.1 |
| | | *Pantoea ananatis* | PNA_18_8S | 99% | 90.71 | 0 | JABEAT010000005.1 |
| | | *Pantoea ananatis* | PNA_18_6S | 99% | 90.71 | 0 | JABEAR010000027.1 |
| | | *Pantoea ananatis* | PNA_18_10S | 99% | 90.71 | 0 | JABEAM010000005.1 |
| | | *Pantoea stewartii* | S301 | 99% | 90.12 | 0 | LIIU01000016.1 |
| | | *Pantoea ananatis* | PA4 | 99% | 91.19 | 0 | JMJK01000013.1 |
| | | *Edwardsiella hoshinae* | NCTC12121 | 99% | 74.64 | 0 | UFXZ01000001.1 |
| | | *Edwardsiella hoshinae* | NBRC 105699 | 99% | 74.64 | 0 | BAUC01000015.1 |
| | | *Dickeya chrysanthemi* | L11 | 98% | 83.23 | 0 | JSYH01000048.1 |
| | | *Dickeya chrysanthemi* | NCPPB 3533 | 98% | 83.2 | 0 | AOOJ01000042.1 |
| | | *Dickeya chrysanthemi* | NCPPB 516 | 98% | 83.2 | 0 | AOOC01000018.1 |
| | **collection** | N/A | | | | | |
| **Pantocin B** | **nr** | N/A | | | | | |
| | **wgs** | *Pantoea agglomerans* | Eh318 | 100% | 100 | 0 | AXOF01000035.1 |
| | | *Pantoea ananatis* | 92-7 | 99% | 98.37 | 0 | JALKKA010000008.1 |
| | | *Pantoea ananatis* | PNA_92_7 | 99% | 98.37 | 0 | JABEBB010000006.1 |
| | **collection** | N/A | | | | | |
| **PNP-1** | **nr** | *Pectobacteriaceae* bacterium | CE90 | 99% | 83.07 | 0 | CP129114.1 |

| | | | | | | | |
|---|---|---|---|---|---|---|---|
| | **wgs** | *Pantoea ananatis* | BRT175 | 100% | 100 | 0 | ASJH01000002.1 |
| | | *Pantoea ananatis* | PANS 99-36 | 100% | 99.88 | 0 | NMZT01000006.1 |
| | | *Pantoea ananatis* | 99-36 | 100% | 99.88 | 0 | JALKLH010000005.1 |
| | | *Pantoea ananatis* | 99-25 | 100% | 99.88 | 0 | JALKLC010000003.1 |
| | | *Pantoea ananatis* | PANS_99_36 | 100% | 99.88 | 0 | JABDZO010000006.1 |
| | | *Pantoea ananatis* | PANS_99_25 | 100% | 99.88 | 0 | JABDZH010000003.1 |
| | | *Pantoea stewartii* subsp. *indologenes* | NCPPB 1562 | 100% | 97.17 | 0 | JADWWO010000020.1 |
| | | *Pantoea stewartii* subsp. *indologenes* | NCPPB 2282 | 100% | 97.17 | 0 | JADWWN010000019.1 |
| | **collection** | N/A | | | | | |
| **PNP-2** | **nr** | *Pantoea agglomerans* | TX10 | 100% | 100 | 0 | MN329808.1 |
| | | *Pantoea agglomerans* | PSV1-7 | 100% | 99.81 | 0 | CP091190.1 |
| | | *Pantoea agglomerans* | CFSAN047153 | 100% | 99.36 | 0 | CP034470.1 |
| | | *Pantoea agglomerans* | CFSAN047154 | 100% | 99.36 | 0 | CP034475.1 |
| | | *Serratia marcescens* | M158-1-1 | 92% | 71.36 | 0 | CP060440.1 |
| | | *Serratia marcescens* | 332 | 91% | 71.52 | 0 | CP021164.1 |
| | | *Enterobacter chengduensis* | WCHECl-C4 | 80% | 69.32 | 0 | CP043318.1 |
| | | *Pectobacterium sp.* | F1-1 | 80% | 67.27 | 0 | CP104733.1 |
| | | *Pectobacterium carotovorum* | 25.1 | 76% | 67.8 | 0 | CP088019.1 |
| | | *Pectobacterium colocasium* | LJ1 | 80% | 67.18 | 0 | CP084032.1 |
| | | *Proteus penneri* | S178-2 | 73% | 66.94 | 0 | CP059690.1 |
| | | *Proteus penneri* | FDAARGOS_874 | 73% | 66.91 | 0 | CP065722.1 |
| | | *Providencia huaxiensis* | WCHPr000369 | 72% | 66.53 | 0 | CP031123.2 |
| | | *Providencia rettgeri* | PROV087 | 72% | 66.49 | 0 | CP059347.1 |
| | | *Providencia rettgeri* | Pre20-95 | 66% | 66.36 | 0 | CP096258.1 |
| | | *Providencia rettgeri* | Res13-Sevr-LER2-35 | 69% | 66.47 | 0 | CP062821.1 |
| | | *Serratia marcescens* | KC049 | 93% | 71.46 | 0 | AP028534.1 |
| | | *Serratia marcescens* | QC13 | 92% | 71.43 | 0 | AP028571.1 |

| | | | | | | |
|---|---|---|---|---|---|---|
| | *Serratia marcescens* | KC034 | 91% | 71.59 | 0 | AP028531.1 |
| | *Serratia marcescens* | KC058 | 93% | 71.34 | 0 | AP028539.1 |
| | *Serratia marcescens* | QC21 | 92% | 71.54 | 0 | AP028580.1 |
| **wgs** | *Pantoea pleuroti* | JZB 2120015 | 100% | 99.9 | 0 | SBFD01000004.1 |
| | *[Curtobacterium] plantarum* (*Pantoea agglomerans*) | RIT-As-4 2 | 100% | 99.86 | 0 | JAPJDK010000002.1 |
| | *Pantoea agglomerans* | CFBP13569 | 100% | 99.86 | 0 | JACYNG010000005.1 |
| | *Pantoea agglomerans* | 4 | 100% | 99.84 | 0 | JPOT02000002.1 |
| | *Pantoea agglomerans* | A2 | 100% | 99.83 | 0 | WSSP01000019.1 |
| | *Pantoea agglomerans* | C1 | 100% | 99.81 | 0 | SMLN01000002.1 |
| | *Pantoea agglomerans* | Pa21-13 | 100% | 99.81 | 0 | JACSXI010000003.1 |
| | *Pantoea agglomerans* | Pa17-5 | 100% | 99.74 | 0 | JACSXM010000002.1 |
| | *Pantoea sp.* | PMG_056 | 100% | 99.65 | 0 | SECZ01000008.1 |
| | *Pantoea agglomerans* | GB1 | 100% | 99.65 | 0 | JYGW01000013.1 |
| | *Pantoea agglomerans* | 62e | 100% | 99.31 | 0 | JACGXI010000002.1 |
| | *Pantoea agglomerans* | 62d | 100% | 99.31 | 0 | JACGXH010000003.1 |
| | *Pantoea agglomerans* | 3 | 100% | 99.2 | 0 | LVHW01000002.1 |
| | *Pantoea vagans* | PaVv1 | 100% | 99.2 | 0 | CEFP01000003.1 |
| | *Pantoea agglomerans* | IG1 | 100% | 99.2 | 0 | BAEF01000002.1 |
| | *Pantoea agglomerans* | MM2021_7 | 100% | 99.19 | 0 | JAFMNV010000002.1 |
| | *Pantoea sp.* | S62 | 96% | 73.14 | 0 | JACVVI010000001.1 |
| | *Pantoea vagans* | Mg1 | 96% | 73.12 | 0 | JAPJYL010000036.1 |
| | *Pantoea vagans* | UBA6298 | 93% | 73.36 | 0 | DJTW01000018.1 |
| | *Pantoea agglomerans* | VRA_MhP_f | 76% | 71.36 | 0 | WKLC01000013.1 |
| | *Pantoea agglomerans* | T6 | 93% | 70.79 | 0 | WSSZ01000009.1 |
| | *Pantoea agglomerans* | Sl1_M5 | 93% | 70.77 | 0 | ADWZ01000022.1 |
| | *Serratia marcescens* | SER00079 | 97% | 70.45 | 0 | JADTQD010000032.1 |
| | *Serratia marcescens* | SER00249 | 97% | 70.41 | 0 | JAEHSS010000001.1 |
| | *Serratia marcescens* | 1145_SMAR | 93% | 71.46 | 0 | JWBL01000131.1 |

| | | | | | |
|---|---|---|---|---|---|
| *Serratia marcescens* | QC05 | 93% | 71.46 | 0 | BQCT01000021.1 |
| *Serratia marcescens* | BM19 | 78% | 71.69 | 0 | JAIWVP010000037.1 |
| *Serratia marcescens* | SER00283 | 92% | 71.41 | 0 | JAFIZV010000023.1 |
| *Serratia marcescens* | 14BL05 | 92% | 71.26 | 0 | BPXK01000009.1 |
| *Serratia marcescens* | AS012323 | 92% | 71.54 | 0 | VKXA01000004.1 |
| *Serratia marcescens* | SRM-1 | 92% | 71.3 | 0 | PXIB01000001.1 |
| *Serratia marcescens* | 907_SMAR | 92% | 71.54 | 0 | JUOM01000128.1 |
| *Serratia marcescens* | SER00255 | 91% | 71.54 | 0 | JAEHSX010000013.1 |
| *Serratia marcescens* | B77-CPSm | 91% | 71.54 | 0 | JAAAME010000003.1 |
| *Serratia marcescens* | M52-CPSm | 91% | 71.54 | 0 | JAAAMB010000001.1 |
| *Serratia marcescens* | PDL100 | 91% | 71.52 | 0 | RCDL01000433.1 |
| *Serratia marcescens* | MS14424 | 93% | 71.17 | 0 | DACTED010000012.1 |
| *Serratia marcescens* | TUM2341 (1) | 43% | 70.94 | 0 | JAUEHN010000078.1 |
| *Serratia marcescens* | TUM2341 (2) | 43% | 70.94 | 0 | BRPU01000078.1 |
| *Serratia marcescens* | KC030 | 43% | 70.94 | 0 | BQAX01000001.1 |
| *Pectobacterium polaris* | ZRIMU1022 | 78% | 67.89 | 0 | JAQRHQ010000001.1 |
| *Pectobacterium aquaticum* | A101-S19-F16 | 75% | 67.88 | 0 | QHJV02000012.1 |
| *Pectobacterium aquaticum* | A105-S21-F16 | 75% | 67.88 | 0 | QHJT02000008.1 |
| *Pectobacterium brasiliense* | CFBP8736 | 75% | 67.88 | 0 | JACDSF010000067.1 |
| *Pectobacterium versatile* | KC01 | 75% | 67.63 | 0 | JAINZP010000007.1 |
| *Pectobacterium versatile* | KC03 | 75% | 67.27 | 0 | JAINZR010000013.1 |
| *Pectobacterium polaris* | Ec-173 | 47% | 66.92 | 0 | SGPT01000035.1 |
| *Proteus penneri* | NCTC12737 | 73% | 66.94 | 0 | UGTQ01000009.1 |
| *Proteus penneri* | JX20 | 73% | 66.94 | 0 | JAEOXK010000010.1 |
| *Proteus penneri* | PR00195 | 73% | 66.94 | 0 | JAEKCB010000009.1 |
| *Proteus penneri* | 1001216B_150713_H11 | 73% | 66.94 | 0 | JADNNB010000033.1 |
| *Proteus sp.* | G2657 | 73% | 66.94 | 0 | JAABMI010000010.1 |
| *Proteus sp.* | G2658 | 73% | 66.94 | 0 | JAABMH010000024.1 |
| *Proteus sp.* | G2659 | 73% | 66.94 | 0 | JAABMG010000014.1 |

| | | | | | |
|---|---|---|---|---|---|
| *Proteus sp.* | G2661 | 73% | 66.94 | 0 | JAABME010000039.1 |
| *Proteus sp.* | G2662 | 73% | 66.94 | 0 | JAABMD010000032.1 |
| *Proteus sp.* | G2663 | 73% | 66.94 | 0 | JAABMC010000045.1 |
| *Proteus sp.* | G2664 | 73% | 66.94 | 0 | JAABMB010000021.1 |
| *Proteus sp.* | G2667 | 73% | 66.94 | 0 | JAABLY010000013.1 |
| *Proteus sp.* | G2670 | 73% | 66.94 | 0 | JAABLW010000018.1 |
| *Proteus sp.* | G2671 | 73% | 66.94 | 0 | JAABLV010000003.1 |
| *Proteus sp.* | G2672 | 73% | 66.94 | 0 | JAABLU010000015.1 |
| *Proteus penneri* | ATCC 33519 | 73% | 66.91 | 0 | PHFJ01000006.1 |
| *Proteus sp.* | G2660 | 73% | 66.91 | 0 | JAABMF010000003.1 |
| *Proteus sp.* | G2665 | 73% | 66.91 | 0 | JAABMA010000029.1 |
| *Proteus sp.* | G2673 | 73% | 66.91 | 0 | JAABLT010000018.1 |
| *Proteus penneri* | MGYG-HGUT-02488 | 73% | 66.92 | 0 | CABMMT010000003.1 |
| *Proteus penneri* | ATCC 35198 | 73% | 66.92 | 0 | ABVP01000025.1 |
| *Proteus penneri* | S333-3 | 73% | 66.87 | 0 | JAMXYA010000009.1 |
| *Proteus sp.* | G2666 | 73% | 66.87 | 0 | JAABLZ010000001.1 |
| *Proteus sp.* | TJ1640 | 73% | 66.81 | 0 | PENY01000018.1 |
| *Proteus penneri* | WFppe127 | 73% | 65.64 | 7.00E-167 | JAPHVR010000010.1 |
| *Providencia sp.* | PROV017 | 68% | 66.49 | 0 | JANBBC010000008.1 |
| *Providencia sp.* | PROV025 | 68% | 66.49 | 0 | JAMPTC010000023.1 |
| *Providencia sp.* | PROV087 | 72% | 66.49 | 0 | JAMPRM010000010.1 |
| *Providencia sp.* | PROV123 | 72% | 66.49 | 0 | JAMPQH010000010.1 |
| *Providencia rettgeri* | ZDHY182 | 68% | 66.49 | 0 | JAFVLM010000004.1 |
| *Providencia sp.* | M-27 | 72% | 66.49 | 0 | JAALBT010000005.1 |
| *Providencia sp.* | PROV032 | 68% | 66.4 | 0 | JAMPSX010000021.1 |
| *Providencia rettgeri* | AHM9C234BI | 62% | 66.36 | 0 | JANGWI010000020.1 |
| *Providencia rettgeri* | 2021GN-00003 | 62% | 66.32 | 0 | ABANRQ020000008.1 |
| *Providencia rettgeri* | 2022EP-00143 | 62% | 66.29 | 0 | ABKGCK020000010.1 |
| *Providencia stuartii* | undefined 2 | 62% | 66.19 | 0 | ABDUAW020000008.1 |

| | | | | | |
|---|---|---|---|---|---|
| *Providencia rettgeri* | Res13-Sevr-LER2-36 | 69% | 66.47 | 0 | JADACH010000164.1 |
| *Providencia rettgeri* | Res13-Sevr-LER2-33 | 69% | 66.47 | 0 | JADACG010000043.1 |
| *Providencia rettgeri* | Res13-Sevr-LER2-34 | 69% | 66.47 | 0 | JADACF010000022.1 |
| *Enterobacter asburiae* | TZW07 | 80% | 70.17 | 0 | JAENNA010000008.1 |
| *Enterobacter asburiae* | TZW10 | 80% | 70.17 | 0 | JAENMX010000008.1 |
| *Enterobacter asburiae* | TZW02 | 80% | 70.1 | 0 | JAENNF010000019.1 |
| *Enterobacter asburiae* | TZW06 | 80% | 70.07 | 0 | JAENNB010000028.1 |
| *Enterobacter asburiae* | TZW08 | 80% | 70.07 | 0 | JAENMZ010000020.1 |
| *Enterobacter asburiae* | TZW01 | 80% | 70.07 | 0 | JAEEAT010000021.1 |
| *Enterobacter cloacae* | ECC445 | 80% | 69.34 | 0 | JAKLRZ010000007.1 |
| *Enterobacter chengduensis* | C2-143-1 | 80% | 69.32 | 0 | SWHP01000001.1 |
| *Enterobacter chengduensis* | WCHECh090071 | 80% | 69.32 | 0 | RWHT01000001.1 |
| *Enterobacter kobei* | EkBL-II-14(1) | 80% | 69.32 | 0 | NEWG01000136.1 |
| *Enterobacter chengduensis* | CIDEIMsCOL9 | 80% | 69.32 | 0 | JZKT01000013.1 |
| *Enterobacter cloacae* | BWH 43 | 80% | 69.32 | 0 | JMUR01000003.1 |
| *Enterobacter chengduensis* | CAL4_1 | 80% | 69.32 | 0 | JASDEE010000001.1 |
| *Enterobacter chengduensis* | C210031 | 80% | 69.32 | 0 | JAMGOE010000037.1 |
| *Enterobacter chengduensis* | C210198 | 80% | 69.32 | 0 | JAMGLI010000001.1 |
| *Enterobacter chengduensis* | C210236 | 80% | 69.32 | 0 | JAMGJY010000001.1 |
| *Enterobacter chengduensis* | HD5030 | 80% | 69.32 | 0 | JAMFWT010000002.1 |
| *Enterobacter chengduensis* | HD7411 | 80% | 69.32 | 0 | JAMFVX010000003.1 |
| *Enterobacter chengduensis* | HD7423 | 80% | 69.32 | 0 | JAMFVV010000002.1 |
| *Enterobacter chengduensis* | HD7427 | 80% | 69.32 | 0 | JAMFVU010000002.1 |
| *Enterobacter chengduensis* | CCBH27266 | 80% | 69.32 | 0 | JALLDI010000001.1 |
| *Enterobacter chengduensis* | AR1284 | 80% | 69.32 | 0 | JAKMNK010000002.1 |
| *Enterobacter chengduensis* | 141186 | 80% | 69.32 | 0 | JAKMKM010000033.1 |
| *Enterobacter chengduensis* | 120076 | 80% | 69.32 | 0 | JAHETZ010000015.1 |
| *Enterobacter chengduensis* | 120062 | 80% | 69.32 | 0 | JAHETQ010000017.1 |
| *Enterobacter chengduensis* | IR5473 | 80% | 69.32 | 0 | JADKYK010000001.1 |

| | | | | | | | |
|---|---|---|---|---|---|---|---|
| | | *Enterobacter chengduensis* | JBBDAAF-19-0140 | 80% | 69.32 | 0 | DAOZVH010000013.1 |
| | | *Enterobacter chengduensis* | JBEHAAB-19-0213 | 80% | 69.32 | 0 | DAOZCO010000003.1 |
| | | *Enterobacter chengduensis* | JBEHABI-19-0050 | 80% | 69.32 | 0 | DAOWRH010000001.1 |
| | | *Enterobacter chengduensis* | undefined 5 | 80% | 69.32 | 0 | DALQKU010000001.1 |
| | | *Enterobacter chengduensis* | ST-414 | 80% | 69.32 | 0 | DAIHWO010000009.1 |
| | | *Enterobacter chengduensis* | M1232 | 80% | 69.32 | 0 | DAHUVW010000026.1 |
| | | *Enterobacter chengduensis* | M07468 | 80% | 69.32 | 0 | DAHJSI010000001.1 |
| | | *Enterobacter chengduensis* | M89665 | 80% | 69.32 | 0 | DAHIGL010000006.1 |
| | | *Enterobacter asburiae* | TUM17577 | 80% | 69.32 | 0 | BQHC01000001.1 |
| | | *Enterobacter bugandensis* | GN03842 | 80% | 68.93 | 0 | LRCL01000087.1 |
| | | *Enterobacter bugandensis* | e1342 | 80% | 68.93 | 0 | FJYI01000004.1 |
| | **collection** | *Pantoea agglomerans* | DC434 | 100% | 99.84 | 0 | |
| | | *Pantoea agglomerans* | SP01220 | 100% | 99.65 | 0 | |
| | | *Pantoea agglomerans* | SP05120 | 100% | 99.36 | 0 | |
| | | *Pantoea agglomerans* | SP05061 | 100% | 99.22 | 0 | |
| **PNP-3** | **nr** | *Pantoea agglomerans* | 3581 | 100% | 100 | 0 | MN807451.1 |
| | | *Pantoea agglomerans* | SS03231 | 100% | 98.95 | 0 | MZ367613.1 |
| | | *Pantoea agglomerans* | SN01080 | 100% | 97.91 | 0 | MN807450.1 |
| | | *Pantoea vagans* | C9-1 | 100% | 95.68 | 0 | CP001894.1 |
| | **wgs** | *Pantoea agglomerans* | Pa31-3 | 100% | 99.4 | 0 | JACSXF010000018.1 |
| | | *Pantoea agglomerans* | VRA_MhP_f | 100% | 99.38 | 0 | WKLC01000003.1 |
| | | *Pantoea agglomerans* | Pa39-3 | 100% | 99.38 | 0 | JACSXB010000018.1 |
| | | *Pantoea agglomerans* | Pa39-14 | 100% | 99.28 | 0 | JACSWY010000018.1 |
| | | *Pantoea agglomerans* | 553Y | 100% | 99.31 | 0 | JAIWYZ010000006.1 |
| | | *Pantoea agglomerans* | 4 | 100% | 98.83 | 0 | JPOT02000004.1 |
| | | *Pantoea sp.* | Ft-CA_14 | 100% | 98.66 | 0 | JALNPM010000001.1 |
| | | *Pantoea sp.* | Ft+CA_17 | 100% | 98.66 | 0 | JALNPL010000001.1 |
| | | *Pantoea agglomerans* | 540Y | 100% | 98.31 | 0 | JAIWZA010000005.1 |
| | | *Pantoea agglomerans* | Pa39-1 | 100% | 95.39 | 0 | JACSXC010000017.1 |

| | | | | | | | |
|---|---|---|---|---|---|---|---|
| | | *Pantoea agglomerans* | Pa31-4 | 100% | 95.37 | 0 | JACSXE010000012.1 |
| | | *Pantoea agglomerans* | Pa39-7 | 100% | 95.37 | 0 | JACSWZ010000010.1 |
| | | *Pantoea agglomerans* | Pa39-21 | 100% | 95.37 | 0 | JACSWX010000006.1 |
| | | *Pantoea agglomerans* | Pa39-23 | 100% | 95.36 | 0 | JACSWW010000011.1 |
| | | *Pantoea sp.* | M_10 | 100% | 91.85 | 0 | VWUL01000006.1 |
| | | *Pantoea sp.* | M_6 | 100% | 91.85 | 0 | VWUH01000008.1 |
| | | *Pantoea sp.* | M_8 | 100% | 91.76 | 0 | VWUG01000012.1 |
| | | *Pantoea agglomerans* | 9Rz4 | 100% | 98.2 | 0 | JANHBZ010000004.1 |
| | **collection** | N/A | | | | | |
| **PNP-4** | **nr** | *Pantoea agglomerans* | B025670 | 100% | 100 | 0 | MT711882.1 |
| | | *Pantoea agglomerans* | DAPP-PG734 | 100% | 99.51 | 0 | OW970315.1 |
| | | *Pantoea deleyi* | LMG24200 | 100% | 99.18 | 0 | CP071405.1 |
| | **wgs** | *Pantoea agglomerans* | CFBP13709 | 100% | 99.6 | 0 | JACYNR010000012.1 |
| | | *Pantoea agglomerans* | CFBP8792 | 100% | 99.6 | 0 | JACYMS010000011.1 |
| | | *Pantoea agglomerans* | CFBP13616 | 100% | 99.54 | 0 | JACYNT010000003.1 |
| | | *Pantoea sp.* | EKM22T | 100% | 99.54 | 0 | JAALFX010000004.1 |
| | | *Pantoea sp.* | EKM21T | 100% | 99.54 | 0 | JAALFV010000007.1 |
| | | *Pantoea agglomerans* | CFBP13566 | 87% | 99.59 | 0 | JACYNI010000031.1 |
| | | *Pantoea ananatis* | 99-33 | 100% | 85.55 | 0 | JALKJN010000017.1 |
| | | *Pantoea ananatis* | PANS_99_33 | 100% | 85.55 | 0 | JABDZN010000014.1 |
| | | *Pantoea dispersa* | JGM112 | 99% | 77.95 | 0 | JAERJL010000061.1 |
| | | *Pantoea dispersa* | JGM106 | 99% | 77.95 | 0 | JAERJK010000042.1 |
| | **collection** | *Pantoea dispersa* | M1657A | 99% | 77.95 | 0 | |
| **PNP-5** | **nr** | *Serratia sp.* | JSRIV006 | 60% | 64.92 | 3.00E-159 | CP074137.1 |
| | | *Yersinia ruckeri* | NVI-10587 | 57% | 64.42 | 1.00E-113 | CP099809.1 |
| | | *Yersinia ruckeri* | NVI-4840 | 57% | 64.42 | 1.00E-113 | CP098703.1 |
| | | *Yersinia ruckeri* | SC09 | 57% | 64.39 | 1.00E-112 | CP025800.1 |
| | | *Yersinia ruckeri* | Barren Creek | 57% | 64.39 | 1.00E-112 | CP133440.1 |

| | | | | | |
|---|---|---|---|---|---|
| *Yersinia ruckeri* | NVI-11065 | 59% | 64.39 | 1.00E-112 | CP098723.1 |
| *Yersinia ruckeri* | NVI-8270 | 59% | 64.39 | 1.00E-112 | CP098694.1 |
| *Yersinia ruckeri* | Big Creek 74 | 57% | 64.39 | 1.00E-112 | CP011078.1 |
| *Yersinia ruckeri* | CSF007-82 | 57% | 64.39 | 1.00E-112 | LN681231.1 |
| *Yersinia ruckeri* | 17Y0189 | 57% | 64.39 | 1.00E-112 | CP084639.1 |
| *Yersinia ruckeri* | 17Y0163 | 59% | 64.39 | 1.00E-112 | CP084641.1 |
| *Yersinia ruckeri* | 17Y0159 | 57% | 64.39 | 1.00E-112 | CP084643.1 |
| *Yersinia ruckeri* | 17Y0155 | 57% | 64.39 | 1.00E-112 | CP084648.1 |
| *Yersinia ruckeri* | 17Y0153 | 57% | 64.39 | 1.00E-112 | CP084650.1 |
| *Yersinia ruckeri* | 16Y0180 | 57% | 64.39 | 1.00E-112 | CP084652.1 |
| *Yersinia ruckeri* | KMM821 | 57% | 64.39 | 1.00E-112 | CP071802.1 |
| *Yersinia ruckeri* | NVI-11050 | 57% | 64.36 | 6.00E-111 | CP099815.1 |
| *Yersinia ruckeri* | NVI-11076 | 57% | 64.36 | 6.00E-111 | CP099808.1 |
| *Yersinia ruckeri* | 17Y0161 | 59% | 64.36 | 6.00E-111 | CP084642.1 |
| *Yersinia ruckeri* | QMA0440 | 57% | 64.33 | 8.00E-110 | CP017236.1 |
| *Yersinia ruckeri* | NVI-11073 | 59% | 64.33 | 8.00E-110 | CP098722.1 |
| *Yersinia ruckeri* | 17Y0157 | 59% | 64.33 | 8.00E-110 | CP084647.1 |
| *Yersinia ruckeri* | NHV_3758 | 59% | 64.29 | 3.00E-108 | CP023184.1 |
| *Yersinia ruckeri* | NVI-492 | 59% | 64.29 | 3.00E-108 | CP099813.1 |
| *Yersinia ruckeri* | NVI-10705 | 59% | 64.29 | 3.00E-108 | CP099805.1 |
| *Yersinia ruckeri* | NVI-10571 | 59% | 64.29 | 3.00E-108 | CP098724.1 |
| *Yersinia ruckeri* | NVI-11267 | 59% | 64.29 | 3.00E-108 | CP098719.1 |
| *Yersinia ruckeri* | NVI-11294 | 59% | 64.29 | 3.00E-108 | CP098716.1 |
| *Yersinia ruckeri* | NVI-1176 | 59% | 64.29 | 3.00E-108 | CP098714.1 |
| *Yersinia ruckeri* | NVI-1292 | 59% | 64.29 | 3.00E-108 | CP098711.1 |
| *Yersinia ruckeri* | NVI-4479 | 59% | 64.29 | 3.00E-108 | CP098710.1 |
| *Yersinia ruckeri* | NVI-4570 | 59% | 64.29 | 3.00E-108 | CP098706.1 |
| *Yersinia ruckeri* | NVI-5089 | 57% | 64.29 | 3.00E-108 | CP098701.1 |
| *Yersinia ruckeri* | NVI-6614 | 59% | 64.29 | 3.00E-108 | CP098697.1 |

| | | | | | | |
|---|---|---|---|---|---|---|
| | *Yersinia ruckeri* | NVI-701 | 59% | 64.29 | 3.00E-108 | CP098695.1 |
| | *Yersinia ruckeri* | NVI-8524 | 59% | 64.29 | 3.00E-108 | CP098691.1 |
| | *Yersinia ruckeri* | 17Y0414 | 59% | 64.29 | 3.00E-108 | CP084635.1 |
| | *Yersinia ruckeri* | 17Y0412 | 59% | 64.29 | 3.00E-108 | CP084637.1 |
| | *Yersinia ruckeri* | YRB | 56% | 64.22 | 5.00E-106 | CP009539.1 |
| **wgs** | *Pantoea sp.* | 1.19 | 31% | 65.85 | 2.00E-56 | MRBS01000001.1 |
| | *Serratia fonticola* | undefined 3 | 66% | 65.04 | 3.00E-167 | CAMKUK010000004.1 |
| | *Serratia sp.* | DD3 | 59% | 65.1 | 3.00E-167 | AYKS02000122.1 |
| | *Serratia sp.* | Res13-Sevr-LER1-36-b | 65% | 65.08 | 3.00E-167 | JADACK010000005.1 |
| | *Serratia sp.* | Res13-Sevr-LER1-36-a | 65% | 65.08 | 3.00E-167 | JADACJ010000084.1 |
| | *Serratia sp.* | 14-2641 | 65% | 65 | 2.00E-164 | LXKR01000066.1 |
| | *Serratia fonticola* | UPMP2124 | 60% | 64.92 | 7.00E-163 | JACNYR010000002.1 |
| | *Serratia fonticola* | UPMP2116 | 60% | 64.92 | 7.00E-163 | JACBJC010000002.1 |
| | *Serratia fonticola* | UPMP2127 | 60% | 64.88 | 4.00E-160 | JACNYO010000006.1 |
| | *Serratia fonticola* | UPMP2128 | 60% | 64.88 | 4.00E-160 | JACBIW010000007.1 |
| | *Serratia fonticola* | UPMP2129 | 60% | 64.88 | 4.00E-160 | JACBIV010000001.1 |
| | *Photobacterium galatheae* | S2753 | 31% | 67.43 | 5.00E-72 | JMIB01000043.1 |
| | *Photobacterium galatheae* | DSM 100496 | 31% | 67.43 | 5.00E-72 | JAGSGC010000018.1 |
| | *Photobacterium salinisoli* | LAM9072 | 20% | 66.08 | 2.00E-51 | QZMS01000011.1 |
| | *Photobacterium sp.* | WH24 | 25% | 66.03 | 7.00E-45 | JAGSOZ010000013.1 |
| | *Photobacterium halotolerans* | P45_P2S44P127 | 18% | 65.99 | 8.00E-44 | WXWV01000336.1 |
| | *Photobacterium sp.* | WH80 | 25% | 65.75 | 1.00E-42 | JAKLTF010000014.1 |
| | *Photobacterium sp.* | WH77 | 25% | 65.75 | 1.00E-42 | JAKLTE010000014.1 |
| | *Photobacterium halotolerans* | DSM 18316 | 30% | 65.87 | 1.00E-42 | AULG01000013.1 |
| | *Photobacterium halotolerans* | P46_P4S1P180 | 18% | 65.75 | 4.00E-41 | WXWW01000156.1 |
| | *Photobacterium sp.* | E2M18 | 25% | 65.62 | 4.00E-41 | JAUOQQ010000038.1 |
| | *Photobacterium halotolerans* | MELD1 | 18% | 65.25 | 6.00E-39 | JWYV01000015.1 |
| | *Photobacterium arenosum* | CAU 1568 | 28% | 64.34 | 6.00E-39 | JACYTP010000020.1 |
| | *Photobacterium halotolerans* | P44_P4S2P179 | 18% | 65.5 | 2.00E-38 | WXWU01000048.1 |

| | | | | | |
|---|---|---|---|---|---|
| *Yersinia ruckeri* | NVI-5983 | 57% | 64.42 | 5.00E-117 | JANATL010000002.1 |
| *Yersinia ruckeri* | NCTC12269 | 59% | 64.42 | 5.00E-117 | JAJIBT010000001.1 |
| *Yersinia ruckeri* | OTH-20-OH-WA-0013 | 59% | 64.42 | 5.00E-117 | ABKTET010000001.1 |
| *Yersinia ruckeri* | FMV-22 | 57% | 64.39 | 6.00E-116 | VDHI01000001.1 |
| *Yersinia ruckeri* | NCTC10476 | 57% | 64.39 | 6.00E-116 | UHJG01000001.1 |
| *Yersinia ruckeri* | NCTC12986 | 57% | 64.39 | 6.00E-116 | UHJF01000001.1 |
| *Yersinia ruckeri* | SCPM-O-B-8085 | 57% | 64.39 | 6.00E-116 | PEHK01000002.1 |
| *Yersinia ruckeri* | 150 | 57% | 64.39 | 6.00E-116 | MKFJ01000012.1 |
| *Yersinia ruckeri* | ATCC 29473 (1) | 57% | 64.39 | 6.00E-116 | JPPT01000001.1 |
| *Yersinia ruckeri* | SCPM-O-B-8298 | 57% | 64.39 | 6.00E-116 | JAQISB010000019.1 |
| *Yersinia ruckeri* | 11-1 | 57% | 64.39 | 6.00E-116 | JANAUW010000002.1 |
| *Yersinia ruckeri* | 11-34 | 57% | 64.39 | 6.00E-116 | JANAUU010000013.1 |
| *Yersinia ruckeri* | 11-5 | 57% | 64.39 | 6.00E-116 | JANAUT010000026.1 |
| *Yersinia ruckeri* | 11-54 | 57% | 64.39 | 6.00E-116 | JANAUS010000011.1 |
| *Yersinia ruckeri* | 11-57 | 57% | 64.39 | 6.00E-116 | JANAUR010000020.1 |
| *Yersinia ruckeri* | 11-73 | 57% | 64.39 | 6.00E-116 | JANAUO010000020.1 |
| *Yersinia ruckeri* | 4025 | 57% | 64.39 | 6.00E-116 | JANAUM010000013.1 |
| *Yersinia ruckeri* | 84015 | 57% | 64.39 | 6.00E-116 | JANAUL010000010.1 |
| *Yersinia ruckeri* | 86020 | 57% | 64.39 | 6.00E-116 | JANAUK010000002.1 |
| *Yersinia ruckeri* | 93010 | 57% | 64.39 | 6.00E-116 | JANAUJ010000002.1 |
| *Yersinia ruckeri* | 99086 | 57% | 64.39 | 6.00E-116 | JANAUI010000008.1 |
| *Yersinia ruckeri* | NVI-10589 | 59% | 64.39 | 6.00E-116 | JANAUH010000010.1 |
| *Yersinia ruckeri* | NVI-11401 | 59% | 64.39 | 6.00E-116 | JANAUF010000011.1 |
| *Yersinia ruckeri* | NVI-1365 | 59% | 64.39 | 6.00E-116 | JANAUD010000007.1 |
| *Yersinia ruckeri* | NVI-2275 | 59% | 64.39 | 6.00E-116 | JANATY010000025.1 |
| *Yersinia ruckeri* | NVI-2909 | 59% | 64.39 | 6.00E-116 | JANATW010000001.1 |
| *Yersinia ruckeri* | NVI-2966 | 59% | 64.39 | 6.00E-116 | JANATV010000017.1 |
| *Yersinia ruckeri* | NVI-2970 | 59% | 64.39 | 6.00E-116 | JANATU010000019.1 |
| *Yersinia ruckeri* | NVI-5635 | 59% | 64.39 | 6.00E-116 | JANATM010000001.1 |

| | | | | | |
|---|---|---|---|---|---|
| *Yersinia ruckeri* | NCTC12266 | 57% | 64.39 | 6.00E-116 | JAJIBR010000001.1 |
| *Yersinia ruckeri* | NVI-11000 | 57% | 64.39 | 6.00E-116 | JAJIBO010000002.1 |
| *Yersinia ruckeri* | RS41 | 41% | 64.39 | 6.00E-116 | CQBN01000003.1 |
| *Yersinia ruckeri* | RuckeriXTk35 | 57% | 64.39 | 6.00E-116 | CCYO01000024.1 |
| *Yersinia ruckeri* | ATCC 29473 (2) | 57% | 64.39 | 6.00E-116 | ACCC01000028.1 |
| *Yersinia ruckeri* | OTH-19-VL-OH-WA-0055 | 57% | 64.39 | 6.00E-116 | ABKTJC010000001.1 |
| *Yersinia ruckeri* | OTH-19-VL-OH-WA-0062 | 59% | 64.39 | 6.00E-116 | ABKTJB010000032.1 |
| *Yersinia ruckeri* | OTH-19-VL-OH-WA-0054 | 59% | 64.39 | 6.00E-116 | ABKTJA010000010.1 |
| *Yersinia ruckeri* | 11/4175-3k | 57% | 64.36 | 2.00E-114 | MECC01000054.1 |
| *Yersinia ruckeri* | 11/4666-4k | 57% | 64.36 | 2.00E-114 | MECB01000033.1 |
| *Yersinia ruckeri* | 12/3871-3K | 57% | 64.36 | 2.00E-114 | MECA01000033.1 |
| *Yersinia ruckeri* | AHL2 | 59% | 64.36 | 2.00E-114 | MEBX01000044.1 |
| *Yersinia ruckeri* | AHL4 | 59% | 64.36 | 2.00E-114 | MEBV01000050.1 |
| *Yersinia ruckeri* | AHL5 | 57% | 64.36 | 2.00E-114 | MEBU01000055.1 |
| *Yersinia ruckeri* | AHL6 | 57% | 64.36 | 2.00E-114 | MEBT01000044.1 |
| *Yersinia ruckeri* | AHL7 | 57% | 64.36 | 2.00E-114 | MEBS01000020.1 |
| *Yersinia ruckeri* | Feb-00 | 57% | 64.36 | 2.00E-114 | MEAK01000046.1 |
| *Yersinia ruckeri* | 00/1445 | 57% | 64.36 | 2.00E-114 | MEAC01000055.1 |
| *Yersinia ruckeri* | 89/3717-10 | 57% | 64.36 | 2.00E-114 | MDZN01000011.1 |
| *Yersinia ruckeri* | 2081 StPb PI | 57% | 64.36 | 2.00E-114 | JAUEHV010000008.1 |
| *Yersinia ruckeri* | 11-31 | 59% | 64.36 | 2.00E-114 | JANAUV010000021.1 |
| *Yersinia ruckeri* | 11-66 | 41% | 64.36 | 2.00E-114 | JANAUP010000049.1 |
| *Yersinia ruckeri* | 11-50 | 62% | 64.36 | 2.00E-114 | JANAUN010000015.1 |
| *Yersinia ruckeri* | NVI-11046 | 57% | 64.36 | 2.00E-114 | JANAUG010000001.1 |
| *Yersinia ruckeri* | NVI-4098 | 57% | 64.36 | 2.00E-114 | JANATR010000001.1 |
| *Yersinia ruckeri* | NVI-4987 | 59% | 64.36 | 2.00E-114 | JANATN010000003.1 |
| *Yersinia ruckeri* | NVI-6390 | 57% | 64.36 | 2.00E-114 | JANATK010000001.1 |

| | | | | | |
|---|---|---|---|---|---|
| *Yersinia ruckeri* | NVI-7989 | 57% | 64.36 | 2.00E-114 | JANATJ010000001.1 |
| *Yersinia ruckeri* | NCTC12270 | 59% | 64.36 | 2.00E-114 | JAJIBU010000002.1 |
| *Yersinia ruckeri* | NCTC12268 | 59% | 64.36 | 2.00E-114 | JAJIBS010000001.1 |
| *Yersinia ruckeri* | OMBL4 | 59% | 64.36 | 2.00E-114 | CPUZ01000014.1 |
| *Yersinia ruckeri* | undefined 4 | 59% | 64.36 | 2.00E-114 | CAMTGJ010000032.1 |
| *Yersinia ruckeri* | IP27752 | 59% | 64.36 | 2.00E-114 | CABIHR010000025.1 |
| *Yersinia ruckeri* | OTH-19-VL-OH-WA-0034 | 59% | 64.36 | 2.00E-114 | ABKTJD010000003.1 |
| *Yersinia ruckeri* | OTH-19-VL-OH-WA-0039 | 57% | 64.36 | 2.00E-114 | ABKTIZ010000035.1 |
| *Yersinia ruckeri* | OTH-19-VL-OH-WA-0037 | 59% | 64.36 | 2.00E-114 | ABKTIX010000035.1 |
| *Yersinia ruckeri* | OTH-20-OH-WA-0016 | 59% | 64.36 | 2.00E-114 | ABKTEY010000049.1 |
| *Yersinia ruckeri* | OTH-20-OH-WA-0036 | 59% | 64.36 | 2.00E-114 | ABKTEX010000001.1 |
| *Yersinia ruckeri* | OTH-20-OH-WA-0017 | 59% | 64.36 | 2.00E-114 | ABKTEW010000002.1 |
| *Yersinia ruckeri* | OTH-20-OH-WA-0052 | 59% | 64.36 | 2.00E-114 | ABKTEV010000001.1 |
| *Yersinia ruckeri* | AQ-22-VL-OH-WA-0002 | 59% | 64.36 | 2.00E-114 | ABKSWW010000066.1 |
| *Yersinia ruckeri* | AQ-22-VL-OH-WA-0001 | 59% | 64.36 | 2.00E-114 | ABKSWU010000002.1 |
| *Yersinia ruckeri* | 05/0297-5eye | 57% | 64.33 | 3.00E-113 | MECI01000079.1 |
| *Yersinia ruckeri* | 07/3342-1k | 57% | 64.33 | 3.00E-113 | MECH01000001.1 |
| *Yersinia ruckeri* | 07/3642-5K-b | 57% | 64.33 | 3.00E-113 | MECG01000001.1 |
| *Yersinia ruckeri* | 07/3828-6E | 57% | 64.33 | 3.00E-113 | MECF01000077.1 |
| *Yersinia ruckeri* | 08/0188-3K | 57% | 64.33 | 3.00E-113 | MECE01000004.1 |
| *Yersinia ruckeri* | 09/0217-5k | 57% | 64.33 | 3.00E-113 | MECD01000087.1 |
| *Yersinia ruckeri* | 14/0125-1k | 57% | 64.33 | 3.00E-113 | MEBZ01000079.1 |
| *Yersinia ruckeri* | AHL1 | 57% | 64.33 | 3.00E-113 | MEBY01000065.1 |
| *Yersinia ruckeri* | AHL3 | 57% | 64.33 | 3.00E-113 | MEBW01000064.1 |
| *Yersinia ruckeri* | 05/0285-1K | 57% | 64.33 | 3.00E-113 | MEAP01000076.1 |

| | | | | | |
|---|---|---|---|---|---|
| *Yersinia ruckeri* | 04/2640-8k | 57% | 64.33 | 3.00E-113 | MEAO01000076.1 |
| *Yersinia ruckeri* | 04/1779 | 57% | 64.33 | 3.00E-113 | MEAN01000083.1 |
| *Yersinia ruckeri* | 04/1749 | 57% | 64.33 | 3.00E-113 | MEAM01000077.1 |
| *Yersinia ruckeri* | Mar-05 | 57% | 64.33 | 3.00E-113 | MEAL01000013.1 |
| *Yersinia ruckeri* | Feb-68 | 57% | 64.33 | 3.00E-113 | MEAJ01000082.1 |
| *Yersinia ruckeri* | 02/0981-4br | 57% | 64.33 | 3.00E-113 | MEAI01000085.1 |
| *Yersinia ruckeri* | 02/0972-2br | 57% | 64.33 | 3.00E-113 | MEAH01000001.1 |
| *Yersinia ruckeri* | 01/0298 | 57% | 64.33 | 3.00E-113 | MEAG01000084.1 |
| *Yersinia ruckeri* | 01/0230-7 | 57% | 64.33 | 3.00E-113 | MEAF01000001.1 |
| *Yersinia ruckeri* | 00/2994 | 57% | 64.33 | 3.00E-113 | MEAE01000103.1 |
| *Yersinia ruckeri* | 00/1793-k2 | 57% | 64.33 | 3.00E-113 | MEAD01000002.1 |
| *Yersinia ruckeri* | 00/0652-K3 | 57% | 64.33 | 3.00E-113 | MEAB01000078.1 |
| *Yersinia ruckeri* | 97/0226-1 | 57% | 64.33 | 3.00E-113 | MEAA01000072.1 |
| *Yersinia ruckeri* | 97/1152-1 | 57% | 64.33 | 3.00E-113 | MDZZ01000079.1 |
| *Yersinia ruckeri* | 96/5134-k | 57% | 64.33 | 3.00E-113 | MDZY01000078.1 |
| *Yersinia ruckeri* | 95/6654-1 | 57% | 64.33 | 3.00E-113 | MDZX01000076.1 |
| *Yersinia ruckeri* | 95/4881-1 | 57% | 64.33 | 3.00E-113 | MDZW01000079.1 |
| *Yersinia ruckeri* | 93/5839-1 | 57% | 64.33 | 3.00E-113 | MDZV01000050.1 |
| *Yersinia ruckeri* | 93/1038-1 | 57% | 64.33 | 3.00E-113 | MDZU01000103.1 |
| *Yersinia ruckeri* | 92/5354-1 | 57% | 64.33 | 3.00E-113 | MDZT01000076.1 |
| *Yersinia ruckeri* | 91/4316 | 57% | 64.33 | 3.00E-113 | MDZS01000039.1 |
| *Yersinia ruckeri* | 91/4311 A1 | 57% | 64.33 | 3.00E-113 | MDZR01000080.1 |
| *Yersinia ruckeri* | 90/4316 | 57% | 64.33 | 3.00E-113 | MDZQ01000082.1 |
| *Yersinia ruckeri* | 90/0961-C9 | 57% | 64.33 | 3.00E-113 | MDZP01000085.1 |
| *Yersinia ruckeri* | 89/4243 | 57% | 64.33 | 3.00E-113 | MDZO01000086.1 |
| *Yersinia ruckeri* | 88/4281-4 | 57% | 64.33 | 3.00E-113 | MDZM01000083.1 |
| *Yersinia ruckeri* | 88/3873 | 57% | 64.33 | 3.00E-113 | MDZL01000085.1 |
| *Yersinia ruckeri* | 88/3837 | 57% | 64.33 | 3.00E-113 | MDZK01000081.1 |
| *Yersinia ruckeri* | 87/3421-SP | 57% | 64.33 | 3.00E-113 | MDZJ01000001.1 |

| | | | | | | |
|---|---|---|---|---|---|---|
| | *Yersinia ruckeri* | 37551 | 57% | 64.33 | 3.00E-113 | JPFO01000016.1 |
| | *Yersinia ruckeri* | NVI-1389 | 59% | 64.33 | 3.00E-113 | JANAUC010000003.1 |
| | *Yersinia ruckeri* | NVI-1660 | 59% | 64.33 | 3.00E-113 | JANAUB010000005.1 |
| | *Yersinia ruckeri* | NVI-2205 | 59% | 64.33 | 3.00E-113 | JANAUA010000018.1 |
| | *Yersinia ruckeri* | NVI-2274 | 59% | 64.33 | 3.00E-113 | JANATZ010000029.1 |
| | *Yersinia ruckeri* | NVI-2775 | 59% | 64.33 | 3.00E-113 | JANATX010000014.1 |
| | *Yersinia ruckeri* | NVI-3779 | 59% | 64.33 | 3.00E-113 | JANATS010000001.1 |
| | *Yersinia ruckeri* | NVI-4507 | 59% | 64.33 | 3.00E-113 | JANATP010000003.1 |
| | *Yersinia ruckeri* | NVI-495 | 59% | 64.33 | 3.00E-113 | JANATO010000010.1 |
| | *Yersinia ruckeri* | NVI-8331 | 59% | 64.33 | 3.00E-113 | JANATI010000007.1 |
| | *Yersinia ruckeri* | NVI-1347 | 59% | 64.33 | 3.00E-113 | JAJIBG010000003.1 |
| | *Yersinia ruckeri* | IP27754 | 59% | 64.33 | 3.00E-113 | CABIHT010000069.1 |
| | *Yersinia ruckeri* | OTH-19-VL-OH-WA-0038 | 59% | 64.33 | 3.00E-113 | ABKTIY010000001.1 |
| | *Yersinia ruckeri* | 11-65 | 57% | 64.29 | 1.00E-111 | JANAUQ010000062.1 |
| | *Yersinia ruckeri* | NVI-1290 | 59% | 64.29 | 1.00E-111 | JANAUE010000001.1 |
| | *Yersinia ruckeri* | NVI-4493 | 59% | 64.29 | 1.00E-111 | JANATQ010000001.1 |
| | *Yersinia ruckeri* | NVI-344 | 59% | 64.29 | 1.00E-111 | JAJIBV010000001.1 |
| | *Yersinia ruckeri* | NVI-10990 | 59% | 64.29 | 1.00E-111 | JAJIBN010000001.1 |
| | *Yersinia ruckeri* | NVI-10974 | 59% | 64.29 | 1.00E-111 | JAJIBM010000001.1 |
| | *Yersinia ruckeri* | NVI-9967 | 59% | 64.29 | 1.00E-111 | JAJIBK010000001.1 |
| | *Yersinia ruckeri* | NVI-3629 | 59% | 64.29 | 1.00E-111 | JAJIBH010000002.1 |
| | *Yersinia ruckeri* | NVI-494 | 59% | 64.29 | 1.00E-111 | JAJIBF010000002.1 |
| | *Yersinia ruckeri* | NVI-3736 | 56% | 64.22 | 2.00E-109 | JANATT010000008.1 |
| **collection** | *Pantoea agglomerans* | 20KB447973 | 100% | 100 | 0 | |

**Supplementary Table 5.** Strains with open reading frame predictions identical to those indicated in Figure 11.

| Group | Species | Strain |
|---|---|---|
| * | *Y. ruckeri* | AHL 1 |
| | *Y. ruckeri* | AHL 3 |
| | *Y. ruckeri* | 00/1793-k2 |
| | *Y. ruckeri* | 01/0230-7 |
| | *Y. ruckeri* | 02/0972-2br |
| | *Y. ruckeri* | 00/2994 |
| | *Y. ruckeri* | 00/1445 |
| | *Y. ruckeri* | 01/0298 |
| | *Y. ruckeri* | 04/1779 |
| | *Y. ruckeri* | 02/0981-4br |
| | *Y. ruckeri* | 04/1749 |
| | *Y. ruckeri* | 04/2640-8k |
| | *Y. ruckeri* | 05/0297-5eye |
| | *Y. ruckeri* | 07/3342-1k |
| | *Y. ruckeri* | 05/0285-1K |
| | *Y. ruckeri* | 07/3642-5K-b |
| | *Y. ruckeri* | 07/3828-6E |
| | *Y. ruckeri* | 11-5 |
| | *Y. ruckeri* | 09/0217-5k |
| | *Y. ruckeri* | 08/0188-3K |
| | *Y. ruckeri* | 11-1 |
| | *Y. ruckeri* | 11-34 |
| | *Y. ruckeri* | 11-50 |
| | *Y. ruckeri* | 11-54 |
| | *Y. ruckeri* | 11-57 |
| | *Y. ruckeri* | 11-73 |
| | *Y. ruckeri* | 11/4175-3k |
| | *Y. ruckeri* | 11/4666-4k |
| | *Y. ruckeri* | 12/3871-3K |
| | *Y. ruckeri* | 14/0125-1k |
| | *Y. ruckeri* | 17Y0153 |
| | *Y. ruckeri* | 17Y0155 |
| | *Y. ruckeri* | 90/4316 |
| | *Y. ruckeri* | 89/4243 |
| | *Y. ruckeri* | 88/4281-4 |
| | *Y. ruckeri* | 95/4881-1 |
| | *Y. ruckeri* | 88/3837 |
| | *Y. ruckeri* | 97/1152-1 |
| | *Y. ruckeri* | 91/4316 |
| | *Y. ruckeri* | 91/4311 A1 |
| | *Y. ruckeri* | 150 |
| | *Y. ruckeri* | 93/1038-1 |
| | *Y. ruckeri* | 97/0226-1 |
| | *Y. ruckeri* | 2081 StPb PI |
| | *Y. ruckeri* | 95/6654-1 |
| | *Y. ruckeri* | 92/5354-1 |
| | *Y. ruckeri* | 89/3717-10 |
| | *Y. ruckeri* | 87/3421-SP |
| | *Y. ruckeri* | 90/0961-C9 |
| | *Y. ruckeri* | 99086 |
| | *Y. ruckeri* | 93/5839-1 |
| | *Y. ruckeri* | 84015 |
| | *Y. ruckeri* | 86020 |
| | *Y. ruckeri* | 37551 |
| | *Y. ruckeri* | AHL5 |
| | *Y. ruckeri* | 93010 |
| | *Y. ruckeri* | AHL6 |

| | | |
|---|---|---|
| | *Y. ruckeri* | AHL4 |
| | *Y. ruckeri* | Feb-68 |
| | *Y. ruckeri* | 96/5134-k |
| | *Y. ruckeri* | Feb-00 |
| | *Y. ruckeri* | 4025 |
| | *Y. ruckeri* | Barren Creek |
| | *Y. ruckeri* | 88/3873 |
| | *Y. ruckeri* | Big Creek 74 |
| | *Y. ruckeri* | NCTC12266 |
| | *Y. ruckeri* | NVI-495 |
| | *Y. ruckeri* | NVI-344 |
| | *Y. ruckeri* | NCTC10476 |
| | *Y. ruckeri* | CSF007-82 |
| | *Y. ruckeri* | NCTC12269 |
| | *Y. ruckeri* | Mar-05 |
| | *Y. ruckeri* | KMM821 |
| | *Y. ruckeri* | NCTC12268 |
| | *Y. ruckeri* | NVI-1347 |
| | *Y. ruckeri* | NVI-1389 |
| | *Y. ruckeri* | NVI-1660 |
| | *Y. ruckeri* | NVI-2205 |
| | *Y. ruckeri* | NCTC12986 |
| | *Y. ruckeri* | NVI-2274 |
| | *Y. ruckeri* | NVI-2775 |
| | *Y. ruckeri* | NVI-3779 |
| | *Y. ruckeri* | NVI-4098 |
| | *Y. ruckeri* | NVI-4507 |
| | *Y. ruckeri* | NVI-6390 |
| | *Y. ruckeri* | NVI-7989 |
| | *Y. ruckeri* | NVI-8331 |
| | *Y. ruckeri* | NVI-11000 |
| | *Y. ruckeri* | NVI-11046 |
| | *Y. ruckeri* | NVI-492 |
| | *Y. ruckeri* | NVI-4479 |
| | *Y. ruckeri* | NVI-4840 |
| | *Y. ruckeri* | NVI-5089 |
| | *Y. ruckeri* | NVI-10587 |
| | *Y. ruckeri* | NVI-11050 |
| | *Y. ruckeri* | NVI-11073 |
| | *Y. ruckeri* | NVI-11076 |
| | *Y. ruckeri* | OTH-20-OH-WA-0052 |
| | *Y. ruckeri* | NVI-5983 |
| | *Y. ruckeri* | SC09 |
| | *Y. ruckeri* | OTH-19-VL-OH-WA-0037 |
| | *Y. ruckeri* | OTH-19-VL-OH-WA-0039 |
| | *Y. ruckeri* | OTH-20-OH-WA-0036 |
| | *Y. ruckeri* | SCPM-O-B-8298 |
| | *Y. ruckeri* | SCPM-O-B-8085 |
| | *Y. ruckeri* | OTH-19-VL-OH-WA-0038 |
| | *Y. ruckeri* | OTH-20-OH-WA-0013 |
| | *Y. ruckeri* | ATCC 29473 (1) |
| | *Y. ruckeri* | ATCC 29473 (2) |
| | *Y. ruckeri* | RuckeriXTk35 |
| | *Y. ruckeri* | OTH-20-OH-WA-0017 |
| ** | *Y. ruckeri* | NCTC12270 |
| | *Y. ruckeri* | AQ-22-VL-OH-WA-0002 |
| | *Y. ruckeri* | NVI-494 |
| | *Y. ruckeri* | 17Y0163 |
| | *Y. ruckeri* | 17Y0412 |
| | *Y. ruckeri* | 17Y0414 |
| | *Y. ruckeri* | NVI-1290 |
| | *Y. ruckeri* | NVI-2275 |

| | |
|---|---|
| *Y. ruckeri* | NVI-2909 |
| *Y. ruckeri* | NVI-3629 |
| *Y. ruckeri* | NVI-4987 |
| *Y. ruckeri* | NVI-2970 |
| *Y. ruckeri* | NVI-2966 |
| *Y. ruckeri* | NVI-3736 |
| *Y. ruckeri* | NVI-5635 |
| *Y. ruckeri* | NVI-8270 |
| *Y. ruckeri* | NVI-9967 |
| *Y. ruckeri* | NVI-10974 |
| *Y. ruckeri* | NVI-11401 |
| *Y. ruckeri* | NVI-10990 |
| *Y. ruckeri* | NVI-10589 |
| *Y. ruckeri* | NVI-1292 |
| *Y. ruckeri* | NVI-701 |
| *Y. ruckeri* | NVI-1176 |
| *Y. ruckeri* | NVI-8524 |
| *Y. ruckeri* | NVI-4570 |
| *Y. ruckeri* | NVI-6614 |
| *Y. ruckeri* | NVI-10571 |
| *Y. ruckeri* | NVI-10705 |
| *Y. ruckeri* | NVI-11065 |
| *Y. ruckeri* | NVI-11294 |
| *Y. ruckeri* | NVI-11267 |
| *Y. ruckeri* | YRB |
| *Y. ruckeri* | OTH-19-VL-OH-WA-0054 |
| *Y. ruckeri* | OTH-19-VL-OH-WA-0034 |
| *Y. ruckeri* | OTH-19-VL-OH-WA-0062 |
| *Y. ruckeri* | OTH-20-OH-WA-0016 |
